# Nanomaterial genotoxicity evaluation using the high-throughput p53-binding protein 1 (53BP1) assay

**DOI:** 10.1371/journal.pone.0288737

**Published:** 2023-09-15

**Authors:** Maelle Fontaine, Eline Bartolami, Marion Prono, David Béal, Magda Blosi, Anna L. Costa, Costanza Ravagli, Giovanni Baldi, Simone Sprio, Anna Tampieri, Ivana Fenoglio, Lang Tran, Bengt Fadeel, Marie Carriere

**Affiliations:** 1 CEA, CNRS, IRIG, SyMMES-CIBEST, Univ. Grenoble Alpes, Grenoble, France; 2 National Research Council, Institute of Science, Technology and Sustainability for Ceramic Materials ISSMC-CNR (Former ISTEC-CNR), Faenza, Italy; 3 Ce.Ri.Col, Colorobbia Consulting S.R.L, Sovigliana-Vinci, Firenze, Italy; 4 Department of Chemistry, University of Turin, Turin, Italy; 5 Institute of Occupational Medicine, Edinburgh, Midlothian, United Kingdom; 6 Institute of Environmental Medicine, Karolinska Institutet, Stockholm, Sweden; VIT University, INDIA

## Abstract

Toxicity evaluation of engineered nanomaterials is challenging due to the ever increasing number of materials and because nanomaterials (NMs) frequently interfere with commonly used assays. Hence, there is a need for robust, high-throughput assays with which to assess their hazard potential. The present study aimed at evaluating the applicability of a genotoxicity assay based on the immunostaining and foci counting of the DNA repair protein 53BP1 (p53-binding protein 1), in a high-throughput format, for NM genotoxicity assessment. For benchmarking purposes, we first applied the assay to a set of eight known genotoxic agents, as well as X-ray irradiation (1 Gy). Then, a panel of NMs and nanobiomaterials (NBMs) was evaluated with respect to their impact on cell viability and genotoxicity, and to their potential to induce reactive oxygen species (ROS) production. The genotoxicity recorded using the 53BP1 assay was confirmed using the micronucleus assay, also scored via automated (high-throughput) microscopy. The 53BP1 assay successfully identified genotoxic compounds on the HCT116 human intestinal cell line. None of the tested NMs showed any genotoxicity using the 53BP1 assay, except the positive control consisting in (CoO)(NiO) NMs, while only TiO_2_ NMs showed positive outcome in the micronucleus assay. Only Fe_3_O_4_ NMs caused significant elevation of ROS, not correlated to DNA damage. Therefore, owing to its adequate predictivity of the genotoxicity of most of the tested benchmark substance and its ease of implementation in a high throughput format, the 53BP1 assay could be proposed as a complementary high-throughput screening genotoxicity assay, in the context of the development of New Approach Methodologies.

## 1. Introduction

Toxicity evaluation of nanomaterials (NMs) is challenging due to their unique properties, especially their small size, large specific surface area and intrinsic absorbance and/or fluorescence, which may lead to assay interference [1]. Some of the currently used toxicity assays may not be adapted to these characteristics as some NMs may interfere with their readout [2–6]. Therefore, an important research effort is currently dedicated to the development or the adaptation of existing assays. Moreover, in the context of new approach methodologies development, high-throughput, cost-effective toxicity assays that could evaluate the safety of new NMs within a short time, using automated technologies and small quantities of NMs are needed [7]. One of the aims of the EU-funded BIORIMA project (Risk management of biomaterials) was to assess the suitability of already-existing toxicity assays to the evaluation of nanobiomaterials (NBMs) (i.e., NMs intended for biomedical applications) and to develop new ones, with the overall goal of constructing a risk management framework for NBMs [8].

Genotoxicity is at the forefront of the toxicity evaluation of new products. Recently, Elespuru et al. proposed a strategy for NM genotoxicity testing, which includes the assessment of i) NM mutagenicity using either the in vitro hypoxanthine-guanine phosphoribosyltransférase (HPRT) gene mutation assay or the mouse lymphoma thymidine kinase (TK+/-) assay, and ii) chromosomal damage using either an in vitro chromosomal aberration test or the in vitro micronucleus assay, with some nano-specific adjustments [9]. Some optional assays are also proposed, both in vitro and in vivo, for instance the comet assay, the transgenic rodent mutation assay, the erythrocyte micronucleus test, the bone marrow chromosomal aberration test [9]. Among these optional assays, the most often used is the comet assay, which is available in high-throughput format [10]. The comet assay has been shown to be prone to NM interference, in particular because NM that have accumulated inside cells may hamper the migration of DNA in the comet tail or inversely may cause additional DNA breaks during the electrophoretic migration as DNA may get in direct contact with NMs accumulated in the cytoplasm [11, 12]. Assays focusing on the detection of DNA repair proteins could also be used as optional assays [13]. These assays are very specific, highly sensitive and they can be easily miniaturized and developed in a high-throughput format. The rationale of such assays is that when DNA is broken, repair proteins are recruited at the vicinity of the damage, some of them forming foci. Immunostaining some proteins involved in the DNA repair complex, then counting foci using fluorescence microscopy or measuring the overall fluorescence of the cell nucleus by flow cytometry, western-in-cell, high content analysis (HCA) or Enzyme-Linked Immunosorbent Assay (ELISA), makes the quantitative evaluation of DNA damage possible [14–16]. These assays are less prone to NM interference than other genotoxicity assays. The only interference that can be expected is from NMs that inherently emit some fluorescence or quench the fluorescence; this interference can be overcome by choosing appropriate antibodies whose fluorescence emission does not match that of the NM being tested. In this category of assays, the most often used DNA damage marker is the histone H2AX, which is phosphorylated when a double strand break occurs in the DNA, leading to the so-called γ-H2AX. H2AX phosphorylation is one of the earliest steps of DNA double-strand break repair [17] and high throughput γ-H2AX assay has been used for screening the genotoxicity of a wide variety of chemicals and NMs [15, 18]. While it has been initially described as a specific marker of DNA double-strand breaks, γ-H2AX is currently considered as a marker of a much broader range of DNA lesions, including DNA single and double-strand breaks, bulky DNA adducts, as well as some DNA replication or transcription blocking lesions [15, 19]. H2AX is also phosphorylated following other cellular events that are not related to DNA damage, for instance when the cell undergoes mitosis, apoptosis or senescence, when cells are exposed to hypotonis stress or when they are transfected or infected by an adeno-associated virus [16, 20–23], sometimes leading to a pattern of pan-nuclear γ-H2AX staining that does not correlate with any DNA damage. Therefore, some instances of γ-H2AX relocalisation may be misinterpreted as being linked to a genotoxic event.

The present study aimed at exploring the applicability of an assay based on the immunostaining of another DNA repair protein forming foci, then foci counting in a high-throughput format, for NBM genotoxicity assessment. Several candidate proteins could be used in this purpose, e.g., Mre11, NBS1, Rad50, Rad51, Rad54, BLM, and BRCA1. When γ-H2AX is recruited near the DNA damage, it triggers immediate recruitment of the p53-binding protein 1 (53BP1) [24], which is an important regulator of the cellular response to double strand breaks and has also been used as a marker of DNA damage [25]. We chose to focus on this protein because it has been described as being recruited on DNA lesions earlier than the other DNA repair proteins, and in the entire population of exposed cell [26]. As for γ-H2AX, two 53BP1 quantification methods can be used, i.e., foci counting or global fluorescence measurement. Each 53BP1 foci corresponds to one double-strand break, i.e., one genotoxic event, while the size of foci, and consequently the fluorescence intensity of each foci, varies depending on the type of DNA lesion and on the kinetics of its repair [19, 21, 27]. Therefore, we believe that foci counting is the most appropriate quantification method. We chose to use automated fluorescence microscopy on a High throughput screening/High content analysis (HTS/HCA) image analysis system for counting 53BP1 foci. From the existing literature, 53BP1 foci appeared brighter, larger, and with less fluorescence background compared to foci from other DNA repair proteins (see for instance, see [16, 26]), and to our knowledge pan-nuclear 53BP1 staining has not been reported (see, for instance [22]). This would facilitate proper identification of 53BP1 foci by the automated imaging system, and this was another reason for choosing 53BP1.

First, we applied the assay to a set of eight acknowledged genotoxic agents listed by the European Centre for the Validation of Alternative Methods (ECVAM) [28], in order to confirm the sensitivity of the system in terms of the used cell line and of the detection of DNA damaging events via automated microscopy. We also tested the assay response to X-rays (1 Gy) because the dose-response relationship between X-ray irradiation and number of double strand breaks formed in DNA is well established, making the assay quantitative [29]. Then, the 53BP1 assay was tested on a panel of twelve different NBMs varying in their chemical composition and physico-chemical properties. Results from the 53BP1 assay were cross-checked with those obtained using the micronucleus assay, also scored via automated microscopy. Finally, since it is widely accepted that NM genotoxicity occurs via indirect mechanisms and among them (although not exclusively) DNA attack by NM-generated reactive oxygen species (ROS) or secondary to inflammation [9, 30], the potency of these NBMs to induce elevation of ROS intracellular levels was also investigated.

## 2. Materials and methods

### 2.1. Chemicals and reagents

Unless otherwise indicated, chemicals and reagents were purchased from Merck Sigma-Aldrich and were >95% pure. The compounds recommended for genotoxicity testing by ECVAM [28] were etoposide (#E1383), methane methylsulfonate (MMS, #129925), hydroquinone (#H9003), taxol (ThermoFisher Scientific, paclitaxel, #P3456), Di-(2-ethylhexyl)phthalate (DEHP, #36735), N-Ethyl-N-Nitrosourea (ENU, #N3385), 3’-azido-3’-deoxythymidine (AZT, #A2169), and aflatoxin B1 (AFB1, #A6636) (S1 Table in S1 File).

### 2.2. Nanomaterials and nanobiomaterials

The NMs and NBMs used in this study belong to three distinct categories, i) metal/metal oxide nanoforms (silver, gold, titanium, iron-based nanoforms), ii) organic nanoforms (carbon-based and solid-lipid nanoparticles) and iii) mineral nanoforms (hydroxyapatites). The rationale supporting the selection of the NBMs here investigated stemmed from the choice of covering multiple compositional classes of NBMs relevant for different intended uses both as medical devices (MD) or Advanced Therapy Medical Products (ATMPs) [8]. Metal/metal oxide nanoforms (silver, gold, titanium, iron-based nanoforms) attracted a strong interest for their potential uses in the field of cancer therapy for theranostic purpose [31, 32]. Several iron-based nanoparticles have been approved for clinical use [33]. Organic nanoforms can be valid susbtitutes of metal-based ones. Lipid-based nanoparticles are currently widely used as carriers of several therapeutic agents and vaccines for their high biocompatibility [34]. Carbon nanoparticles show promise as photothermal agents for cancer therapy and as carriers of antimicrobial peptides [35]. Finally, mineral nanoforms (biomineralised hydroxyapatites) are ideal candidates for tissue engineering application, mimicking natural bone formation process [36]. Details on the tested materials are provided as Supplementary Information. Some of them were purchased from Sigma Aldrich (hydroxyapatite, HA1) while others were supplied by the EU Joint Research Center (JRC), i.e., titanium dioxide nanoparticles (TiO2, NM101), silver nanoparticles (Ag NP, NM300K), multi-walled carbon nanotubes (MWCNT, NM400) and zinc oxide nanoparticles (ZnO NPs, NM110) [37], the latter having been investigated separately from the others because it was not initially included in the panel of tested NMs. Other NBMs were supplied by industrial partners from BIORIMA. Colorobbia Consulting s.r.l. (Firenze, Italy) supplied Fe3O4 nanoparticles coated with a block copolymer containing two polymeric units polyethyleneglycol and poly (lactic-co-glycolic acid) (Fe3O4-PEG-PLGA, termed Fe3O4 in this article [38, 39]), gold nanoparticles and gold nanorods (Au NPs and Au NRs [40–43]). Nanovector S.r.l. produced solid-lipid nanoparticles (SLN1 and SLN2 [44]), and Finceramica S.p.a. produced hydroxyapatite-collagen-based scaffolds (HAsc [45, 46]). Other NBMs were produced by BIORIMA academic partners, i.e., silver nanoparticles coated with hydroxyethylcellulose (Ag-HEC [47, 48]), hydroxyapatite powder (HA2), hydroxyapatite doped with iron (FeHA [49, 50]), produced by ISSMC-CNR [51–53]; carbon nanoparticles coated with polyethyleneglycol (CNP-PEG) produced by University of Torino. These NBMs have been conceived for application as drug delivery agents and/or for in vivo imaging, biosensing or therapy (Au NPs, Au NRs, Ag-HEC, Fe3O4-PEG-PLGA, FeHA, HA, CNP-PEG, SLNs), for tissue regeneration (HA2, FeHA, HAsc) or for coating of implants or wounds (Ag-HEC, HA, FeHA). They are considered as bioinert (Au NP, Ag NP, CNP-PEG), bioactive (Fe3O4-PEG-PLGA, HA, SLN) or Biomimetic/Bioresorbable/Stimulating specific cellular responses at molecular level (FeHA). All the NMs and NBMs were evaluated for endotoxin content using the chromogenic endpoint limulus amebocyte lysate (LAL) assay as previously described [54], results are reported in S2 Table in S1 File. Some NBMs interfered with the LAL assay, these were tested using human monocyte-derived macrophages (HMDM) as described previously [55]; HMDMs were not activated by these NBMs.

### 2.3. Handling of NBMs

When NBMs were supplied as powder, they were dispersed using the generic Nanogenotox dispersion protocol [56], i.e., by pre-wetting in ethanol, then diluting in sterile-filtered 0.05% bovine serum albumin (BSA) at the concentration of 2.56 mg/mL (approximate volume of 6 mL), in a scintillation vial, then dispersed via high energy probe sonication with an energy input of 3.136 MJ/m3, which corresponds in our setup to 16 min of sonication at 20% amplitude (Vibracell 75043, Bioblock Scientific) [57]. Immediately after sonication, NBM suspensions were diluted to the hundredth either in ultrapure water (for physico-chemical characterization) or in complete cell culture medium (for physico-chemical characterization and cell exposure). NBMs provided as suspensions were dispersed by vigorous vortexing, then directly diluted in cell culture medium. All of them were immediately analyzed for their hydrodynamic diameter and zeta potential using a Nano series Zetasizer (Malvern), as well as after 24 h of incubation at 37°C, 5% CO_2_.

### 2.4. Cell culture and exposure

The human colorectal carcinoma cell line HCT116 was purchased from the European Cell Culture Collection (ECACC, Salisbury, UK) and was used from passage 12 to passage 30. Cells were grown in McCoy’s 5a medium to which was added 50 U/mL of penicillin, 50 μg/mL streptomycin and 10% (v/v) fetal bovine serum (FBS), at 37°C, 5% CO_2_ in a humidified atmosphere and passed twice a week. They were checked for mycoplasma contamination twice per month. For toxicity experiments, they were seeded at 20 000 cells per well (WST-1 assays and DHR123 assay), 5 000 cells per well (53BP1 assay) or 3 000 cells per well (micronucleus assay) in 96-well plates. Reference genotoxins were dissolved in DMSO to the concentration of 1 M (ENU), 100 mM (MMS, hydroquinone, AZT), 10 mM (Etoposide, Taxol, DEHP) or 1 mM (AFB1). In the WST1 assay, cells were exposed to 0–1 mM of these genotoxins or to 0–100 μg/mL of NBMs. In all other assays, cells were exposed to the highest concentration that did not affect cell viability as estimated via the WST-1 assay (Cmax), half of this concentration and one fifth of this concentration. Regarding NBMs that did not affect cell viability, we chose to test them at 10, 25 and 50 μg/mL. These concentration were chosen so as to avoid assay interference of the NBMs [13, 58] and in order to test the genotoxicity of most of the NBMs at comparable concentration, given their distinct impact on cell viability. X-ray irradiations were performed on a CIX2 irradiator (X Strahl Life Sciences, United Kingdom) performing at 250 kV. The applied dose (1 Gy, representative of a dose used in radiotherapy) was controlled thanks to a Unidos^®^ E dosimeter (PTW, Freiburg, Germany).

### 2.5. WST-1 assay

After cell exposure, exposure medium was discarded and 100 μL of a WST-1 solution (Roche, Basel, Switzerland) diluted to the tenth in cell culture medium was added to each well. The plates were incubated for 90 min at 37°C then absorbance was measured at 450 nm and corrected for background absorbance at 690 nm. In the experiments where cells were exposed to NBMs, to avoid any optical interference of the NMs with the assay, the plates were centrifuged and 50 μL of supernatant was transferred to a clean plate before absorbance measurement. Amine-functionalized polystyrene NPs (PS-NH2, Merck #L0780) were used as positive control (100 μg/mL, 24 h).

### 2.6. 53BP1 assay

**Fig 1:**
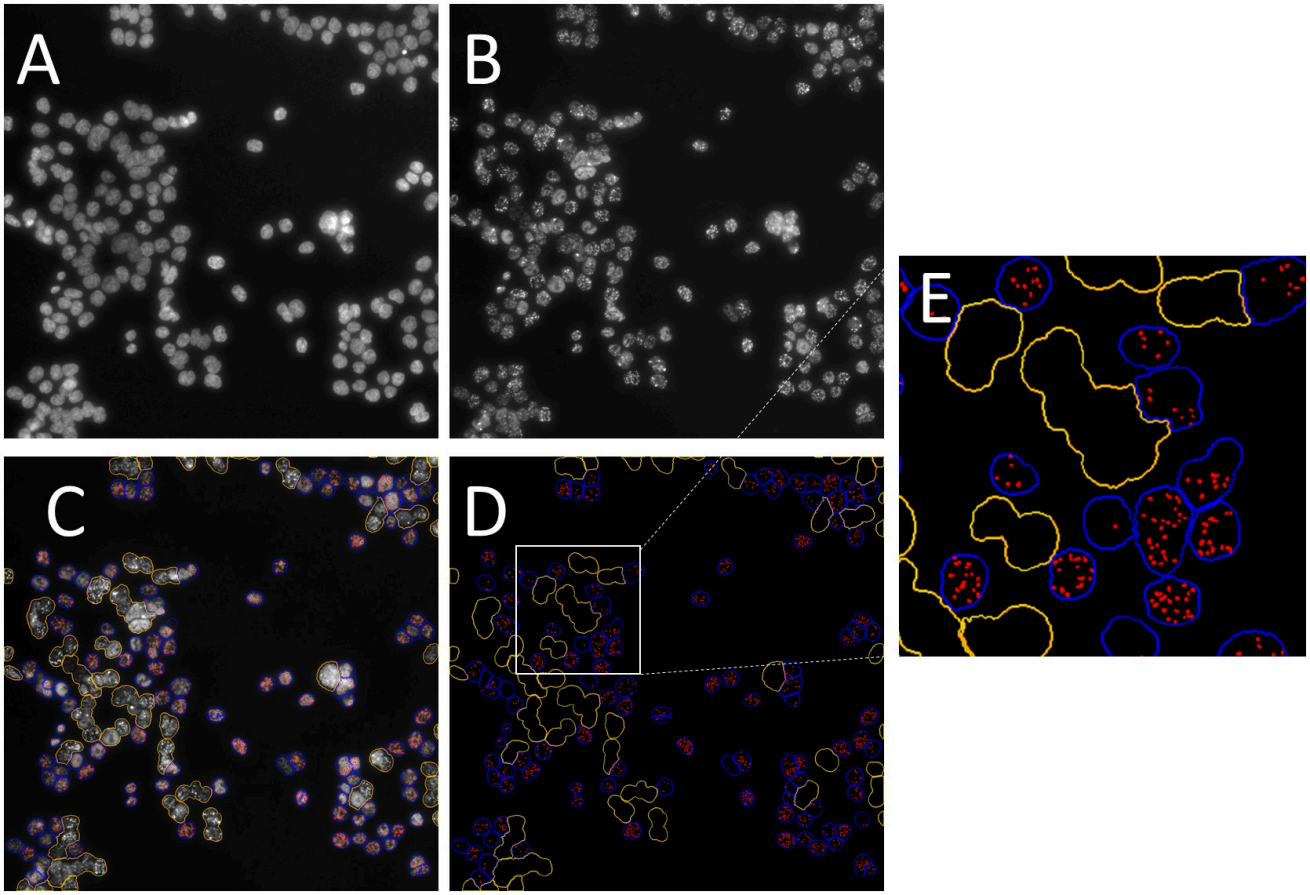
High throughput analysis of 53BP1 foci using the CellInsight CX5 automated imagining and analysis system. Cells were exposed for 24 h to 80 μM of MMS, then fixed and immunostained for 53BP1 foci. Nuclei were counterstained using Hoechst 33342. Fluorescence image of cell nuclei (A), 53BP1 foci (B) are acquired. Then, segmentation is performed using the HCS studio software, which selects nuclei that will be analysed, those that must not be considered, and counts 53BP1 foci in the selected nuclei (represented in blue, yellow and red, respectively, in D and E).

After incubation for 24 h with genotoxins, NBMs or positive controls (etoposide, 50 μM and (CoO)(NiO) nanoparticles, <150nm, Merck #634360, 20 μg/mL), cells were immunostained for 53BP1 using a previously optimized protocol [59]. Briefly, cells were fixed with 4% formaldehyde, pH 7.4, permeabilized with 0.2% triton X-100 for 15 min at room temperature then washed three times with PBS containing 3% bovine serum albumin (BSA) (washing buffer). They were then incubated with an anti-human 53BP1 polyclonal antibody (Abnova, PAB12506, dilution 1/1000, Clinisciences, Nanterre, France) for 1 h at room temperature under mild agitation, rinsed three times for 5 min with washing buffer and incubated for 1 h at room temperature with goat anti-rabbit IgG-Atto488 (Merck #18,772, dilution 1/2000, St. Louis, MO, USA). They were rinsed three times with washing buffer containing 0.2% triton X-100 and counterstained with 0.3 μg/mL Hoechst 33342 for 30 min at room temperature. Each well was then washed three times with PBS, and plates were stored at 4°C in the dark until analysis using a CellInsight CX5 High Content Screening automated imaging and image analysis system (Thermo Fisher Scientific). The workflow for image analysis is depicted in Fig 1. In each well, the automated microcope captures an image of cell nuclei based on the Hoechst 33342 fluorescence (Fig 1A) and then an image of 53BP1 foci based on Atto488 fluorescence (Fig 1B). Then, image segmentation is performed by the HCS studio^™^ so ftware: cell nuclei that are appropriate for 53BP1 foci counting are selected, (they are delineated in blue in Fig 1C–1E, while excluded nuclei are delineated in yellow). Nuclei are selected based on their size and their circularity, thereby excluding fractioned nuclei of apoptotic cells or nuclei from damaged cells, and also nuclei from cells that are too close to one another (i.e., these nuclei appear to be too large), hampering their proper identification and analysis. Nuclei that are on the image edges are also excluded. Within the selected nuclei, HCS studio^™^ identifies 53BP1 foci (depicted in red in Fig 1C–1E) as brighter and smaller spots and counts them. The thresholds for the identification of 53BP1 foci are fixed by the operator based on his informed experience and on the values that are expected in control cells (both unexposed cells and cells exposed to the positive control). An example is reported in Fig 1D, where delineations of selected/excluded nuclei and 53BP1 foci are shown. Fig 1E is a higher magnification image of the white square region of Fig 1D.

Two thousand five hundred cells were analysed per condition, i.e., 500 cells per well in 5 replicate wells. The whole experiment was repeated three times independently, which led to 53BP1 foci counting in 7500 cells per condition. Results are expressed as average numbers of 53BP1 foci per cell nucleus and the reported results are the mean values ± standard deviation of the 15 values obtained from the 5 replicate wells and 3 independent experiments. Note that this assay can also be implemented in a low throughput format using fluorescence microscopy imaging and either manual or semi-automated image analysis using open source image analysis softwares such as image J. Several suppliers propose automated imaging and image analysis systems that make possible high throughput foci counting and data analysis. Some of them are very sofisticated, offering confocal imaging and 3D reconstructions, but for the 53BP1 assay proposed in this article, the simplest system (CellInsight CX5, Thermo Fisher Scientific) is sufficient.

### 2.7. Micronucleus assay

After incubation for 24 h with the genotoxins or NBMs, the genotoxin and NBMs were discarded, and cells were rinsed with PBS before being incubated for 28 h with 4 μg/mL cytochalasin B prepared in complete cell culture medium. At the end of this exposure period, cells were rinsed with PBS then fixed with 4% paraformaldehyde for 15 min at room temperature. Nuclei were stained with 0.3 μg/mL Hoechst 33342 for 30 min at room temperature, then cells were rinsed three times with PBS and stored at 4°C until analysis using a CellInsight CX5 High Content Screening automated imaging and analysis system (Thermo Fisher Scientific). One thousand cells per well were analyzed, on average 25% of them were binucleated. This low rate of binucleated cells may be explained both by the low cell seeding density that would reduce cell proliferation rate, by the toxicity of cytochalasin B and/or by the rejection of some binucleated cells by the HCS Studio^™^ algorithm due to overlapping nuclei or inappropriate shape of binucleated cells. When considering that we used 5 replicates per experiment and three independent experiments per tested substance, this led to counting micronuclei in 3750 binucleated cells per condition, while the OECD TG487 guideline recommends counting 2000 binucleated cells per condition. Mitomycin c (500 ng/mL, exposure for 24 h) was used as positive control. Results are expressed as % micronucleus frequency in targeted cells, i.e., binucleated cells, and reported as the mean ± standard deviation of the 15 values obtained from the 5 replicate wells from each of the 3 independent experiments.

### 2.8. DHR123 assay

Reactive oxygen species (ROS) were quantified using the dihydrorhodamine 123 (DHR123) dye. Cells were incubated with 1 μM of DHR123 prepared in PBS for 45 min at 37°C. Then, they were rinsed with PBS and exposed to NBM dilutions, prepared in complete cell culture medium. Fluorescence was measured just after exposure then after 30 min, 1 h, 3 h, 5h and 24 h of exposure (λexcitation / λemission 480 / 530 nm). Tert-butyl hydroperoxide (TBHP, 250 μM) was used as positive control. Reported results are the mean values ± standard deviation of the 15 values obtained from the 5 replicate wells from each of the 3 independent experiments.

### 2.9. Statistical analysis

Experiments on cells were reproduced three times independently (n = 3), with 5 technical replicates per independent experiment. As assumption for normality and homoscedasticity of data could not be verified due to too low number of independent replicates, non-parametric assays were used for statistical significance assessment, i.e., Kruskall–Wallis test followed by pairwise comparison using Mann-Whitney test. These tests were performed using Graphpad Prism (v. 7.02).

## 3. Results and discussion

### 3.1. The HCT116 cell line and 53BP1 assay for genotoxicity assessment

#### 3.1.1. Choice of the cell line for the 53BP1 assay development

In the present study, we optimised the 53BP1 assay using the HCT116 cell line. The Organisation for Economic Cooperation and Development (OECD) recommends the use of some specific cells lines for genotoxicity assessment in a regulatory context, which are V79, CHL, L5178Y, CHO or TK6 cells, because of their p53 status, genetic stability and DNA repair capacity. Some of these cell lines grow in suspension (L5178Y, TK6), which makes them unsuitable for automated microscopy assays. The other cell lines are not from human origin, and they show a fibroblast or ovary cell morphology and phenotype, which is different from that of intestinal epithelial cells. Nanomaterial are internalized by cells mainly via endocytosis [60, 61]. Therefore, their capacity to internalize NPs is governed by the cell’s endocytic capacity, which differs depending on the origin of the cell line [62]. One of the main functions of intestinal epithelial cells is to absorb nutrients, consequently they are equiped with cell membrane transporters that ensure their absorptive capacity, and they are also endocytosis- and transcytosis-competent, which has been suggested as conferring them the capacity to internalize optimally ~50 nm NPs, but also NP agglomerates with diameter 100–500 nm [63, 64]. Conversely, themain role of fibroblasts is to maintain the structure of tissues via extracellular matrix production and sec retion; their structure and morphology is rather adapted to achieve this goal [65]. Therefore, their ability to uptake NPs or agglomerates of NPs would be lower than that of epithelial intestinal cells. For these reasons, we considered that it would be more suitable to use a human-derived, epithelial intestinal cell line for developing the 53BP1 assay in the frame of the BIORIMA project. Among all human colon-derived cells used for genotoxicity assessment, HT-29, Caco-2 and HCT-116 are the most frequently used. Notably, among these cell lines, only HCT-116 expresses non-mutated p53, which is an important mediator of genomic stability [66]. Even if these cells are from cancerous origin, hold mutations at codon 13 (KRAS, which is a proto oncogene) and overexpresses CBS gene, which should be avoided when assessing genotoxicity, their p53 status makes them better models than Caco-2 and HT-29 cell lines for genotoxicity testing. Moreover, HCT116 cells have high NM endocytosis capacity [62], which would ensure that NMs and NBMs are internalized in sufficient amounts to express their DNA damaging potential. Still, such undifferentiated intestinal cancer cells would not reflect the reality of NBM accumulation in non-malignant human epithelial intestinal cells, which probably do not hold the same endocytic capacities due to their differentiation, as demonstrated earlier in cell lines [67]. Therefore, results obtained via the 53BP1 assay on this specific cell line would need to be confirmed using a more relevant exposure scenario and cell model, such as one of those recommended in OECD guidelines, before a definitive conclusion is reached. Moreover, some potentially genotoxic substances need to be metabolized to express their genotoxic potential, therefore, it would be important to confirm the results on a cell line that expresses sufficient amounts of Phase I and Phase II metabolic enzymes, such as a hepatocyte cell line, for instance HepG2 or HepaRG^™^ cells. Finally, the reader should bear in mind that this study is a proof-of-concept of the applicability of the 53BP1 assay for genotoxicity testing of NMs and NBMs, and that it is not intended to prove that the tested NMs and NBMs are safe.

#### 3.1.2. Choice of benchmark genotoxic substances

First, the accuracy of the 53BP1 assay was assessed by testing the response of HCT116 cells to a series of known genotoxic substances, in order to benchmark the assay. These genotoxins were chosen among the substances listed by ECVAM for assessment of new genotoxicity test performance [28]. They are reported in S1 Table in S1 File. Most of these chemicals belong to ECVAM group 1 substances, i.e., mutagenic carcinogens that should lead to positive outcome in in vitro genotoxicity assays. Seven substances from this group were tested, showing distinct modes of genotoxic action, i.e., DNA alkylating agents (methane methylsulfonate–MMS-, N-ethyl-N-nitrosourea -ENU- and cyclophosphamide), two aneugens (hydroquinone and taxol), a topoisomerase II inhibitor (etoposide), a clastogen inducing replication stress (azidothymidine -AZT-) and a substance producing DNA adducts upon metabolisation by cytochrome P450 [68] (aflatoxin B1 -AFB1-, which is metabolized to to AFB1-8,9-epoxide) (S1 Table in S1 File). Then, we tested one substance from ECVAM group 2, i.e., a well-established and classically-used non DNA damaging agent, which was 2-deoxy-D-glucose. In addition, we tested four substances belonging to ECVAM group 3, i.e., substances that should show a negative outcome in in vitro genotoxicity assays but that were previously reported to induce DNA damage in some assays, often at high concentration. These substances are di-2(ethyl hexylphtalate) (DEHP), eugenol, urea and propyl gallate (PG). Since double strand break formation due to exposure to chemical agents most of the time depends on the progression of cells through the S-phase of the cell cycle, cell response to these substances was tested after 24 h of exposure, which is the approximate doubling time of HCT116 cells. The genotoxic impact of a 1 Gy of X-ray irradiation of the HCT116 cell line was also tested, immediately after the irradiation because it is known to directly induce double strand breaks in the DNA that are rapidly repaired [29]. Moreover, the kinetics of their repair was assessed, also using the 53BP1 assay.

#### 3.1.3. Impact of benchmark genotoxic substances on cell viability

**Fig 2:**
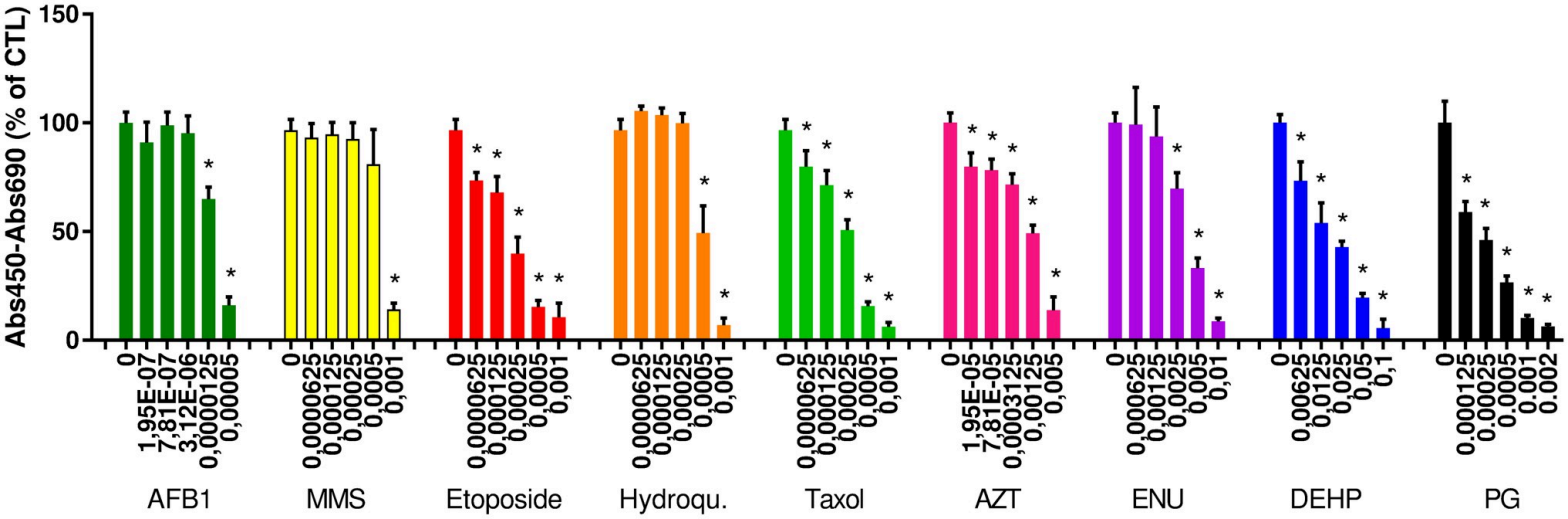
Viability of cells exposed to model genotoxic substances. Cell viability was evaluated using the WST-1 assay after 24 h of exposure to aflatoxin B1 (AFB1), methyl methanesulfonate (MMS), etoposide, hydroquinone (Hydroqu.), taxol, azidothymidine (AZT), N-nitroso-N-ethylurea (ENU), di-2(ethyl hexylphtalate) (DEHP) or propyl gallate (PG). The concentrations of these compounds are expressed in M (x-axis). Depicted are the mean values ± standard deviation of three independent experiments with five replicates per experiment (n = 15).

As a prerequisite to genotoxicity assessment, the 13 test substances were first tested for their cytotoxicity since genotoxicity should not be assessed at concentrations that highly affect their viability [13] (Fig 2). To do so, we used the WST-1 assay, which measures the cleavage of WST-1 tetrazolium salt by cellular dehydrogenases to form the dark red formazan. This assay is classically used as a proxy for cell viability, although it measures cell dehydrogenase activity, which rather reflects the cell number [69]. Therefore, it measures both cell proliferation, cell loss and cell death [70]. It has been described previously to show similar performance as other classically-used cytotoxicity assays that are based on lysosomal integrity (neutral red assay), cell membrane integrity evaluation (lactate dehydrogenase assay) and resazurin reduction (Alamar blue assay), using a panel of six NMs, tested on 12 cellular models [71]. The same has been concluded when comparing its response with that of the lactate dehydrogenase assay on a panel of 23 NMs and 10 cell lines [72]. Moreover, it shows low interference with NMs with minimal adaptation of the protocol [4]. Therefore, it was used here to determine the highest concentration of each compound to be used for genotoxicity testing, chosen to be related to the highest concentration that did not impair cell viability (Cmax). Among the 13 tested substances, 4 substances did not induce any reduction of cell viability up to 10 mM, which were cyclophosphamide, 2-deoxy-D-glucose, eugenol and urea (not shown). Therefore, these substences were excluded from the genotoxicity assessment. For all other substances, the obtained Cmax are reported in S1 Table in S1 File; i.e., 3 μM (AFB1), 500 μM (methyl methanesulfonate, MMS), <50 μM (etoposide), 250 μM (hydroquinone), <62.5 μM (taxol); <20 μM (azidothymidine, AZT); 1.25 mM (N-nitroso-N-ethylurea, ENU), <6.25 mM (di-2(ethyl hexylphtalate), DEHP); <125 μM (propyl gallate, PG).

Based on these values, genotoxicity was tested at concentrations slightly lower than the Cmax, then half and one fifth of this concentration (S1 Table in S1 File). We chose the highest tested concentration as being slightly lower than the Cmax in order to avoid any misinterpretation of 53BP1 foci that could potentially result from apoptotic cells, but also because cells were seeded at lower density for the 53BP1 assay, compared to the WST-1 assay, and therefore were more sensitive to the toxic substances, as already observed by others (see, for instance [73]). These three concentrations were tested in order to identify any increase of genotoxic damage that would be related to increased genotoxin concentration, although three concentrations are not sufficient to strictly define a dose-response relationship [74]. For hydroquinone, lower concentrations were tested in the 53BP1 assay, because at concentrations close to the Cmax, the cells were so loosely attached to the plate that they were lost during the immunolabelling procedure. This ability of hydroquinone to affect cell adhesion has been already described elsewhere [75], which explains why cells were so loosely attached to the wells. One recommendation would be to combine several cytotoxicity assays in order to have a better view of the substance concentrations to be tested in the 53BP1 assay, or to systematically assess the genotoxicity at a much broader range of concentrations and to incude an estimation of cytotoxicity directly within the genotoxicity assay, for instance via counting cell nuclei in the whole wells before the segmentation and foci counting. This would ensure that the range of tested concentration is better adapted to genotoxicity assessment, although substances that weaken cell adhesion in vitro cannot be considered as impairing cell viability.

#### 3.1.4. Response of benchmark genotoxic substances in the 53BP1 assay

**Fig 3:**
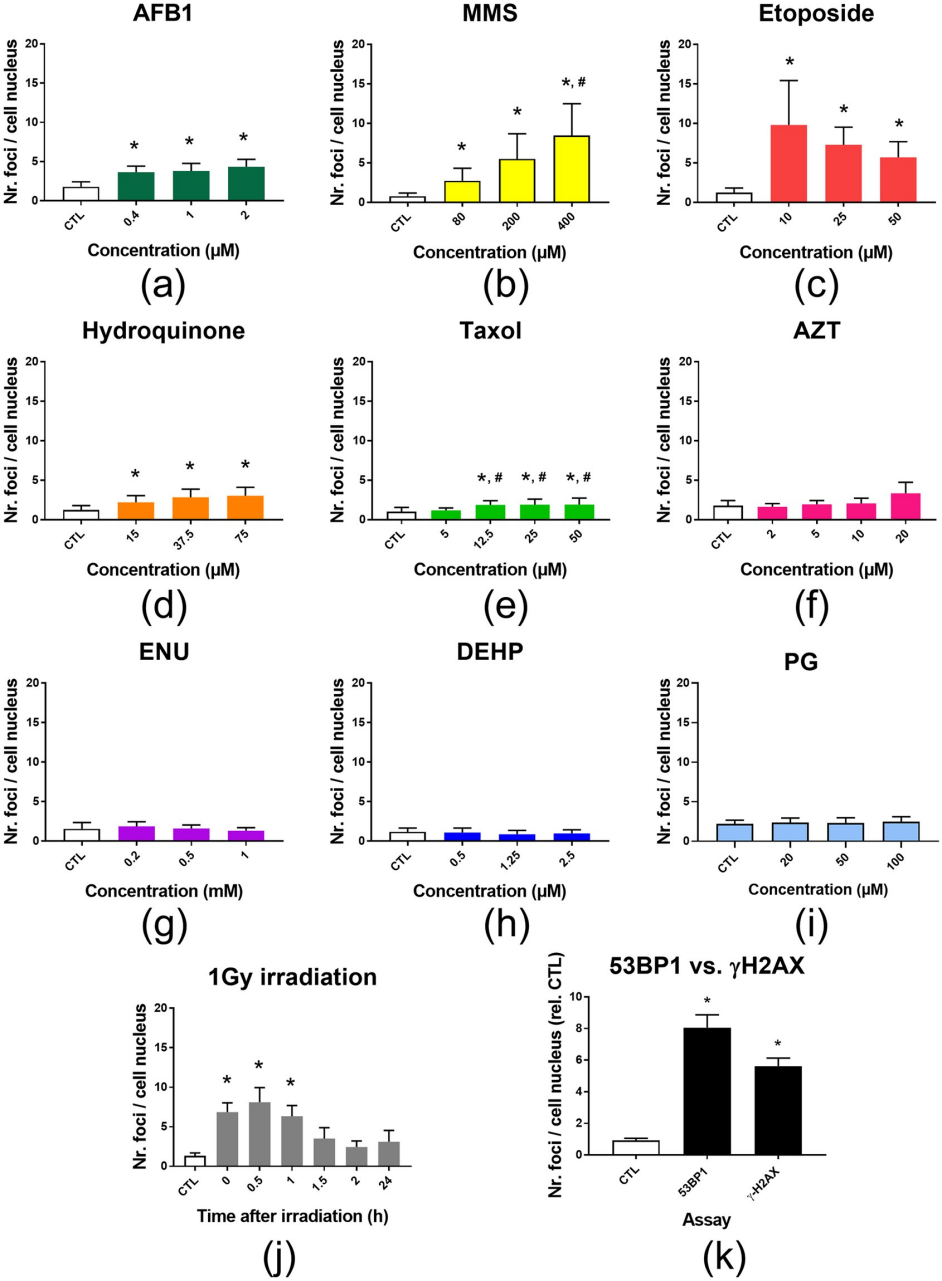
53BP1 assay for genotoxicity assessment of model genotoxicants. HCT116 cells were exposed to (a) aflatoxin B1, (b) methyl methanesulfonate, (c) etoposide, (d) hydroquinone, (e) taxol, (f) azidothymidine, (g) N-nitroso-N-ethylurea, (h) Di(2ethyl hexyl)phthalate, (i) propyl gallate for 24 h and then fixed or to (j) a 1 Gy X-ray irradiation and then fixed 30 min, 1 h, 1 h 30, 2 h or 24 h after irradiation. Then, the 53BP1 assay results was compared to that of γ-H2AX assay, on cells exposed to 1 Gy X-ray irradiation, 30 min after irradiation (k). All these samples were immunostained for 53BP1 (or γ-H2AX) foci, and foci were counted in each cell nucleus using automated fluorescence microscopy. Depicted are the mean number of foci per cell nucleus ± standard deviation of 3 independent experiments with 5 replicates per experiment (n = 15). Statistical significance: *p<0.05, exposed versus control; ^#^p<0.05, 400 μM vs. 80 μM (MMS) or 12.5 μM, 25 μM or 50 μM vs. 5 μM (taxol).

The response of HCT116 cells to the test substances in the 53BP1 assay, at the range of concentration chosen based on the WST-1 assay, is reported in Fig 3, while results obtained in a larger range of tested concentrations are reported in S1 Fig in S1 File. Typical images of cells with 53BP1 foci are shown in S2 Fig in S1 File.

Significant genotoxicity was observed for at least one of the tested concentrations of AFB1, MMS, etoposide, hydroquinone and taxol, when compared to the control (unexposed cells). For all the observed genotoxic substances, the number of foci decreased and became non statistically significant at cytotoxic concentrations (S1 Fig in S1 File), reflecting the loss of damaged cells during the 53BP1 staining procedure, as the cell density was also observed to decrease at these concentrations (not shown). Significant increase of 53BP1 foci counts was observed when concentrations of MMS and taxol increased, with statistical significance at 80 μM compared to 400 μM of MMS, and at 5 μM compared to 12.5 μM, 25 μM or 50 μM of taxol. Still, it would be necessary to test a higher number of concentrations of these substances in order to robustly demonstrate that there is a dose-response relationship. AZT, DEHP and PG did not induce any significant DNA damage, at any of the tested concentration, even the cytotoxic ones (S1 Fig in S1 File). Absence of genotoxic response of DEHP and PG were expected, since these compound are non-genotoxic carcinogens from group 3 in the ECVAM lists. Regarding AZT, its genotoxic mode of action is via triphosphorylation and incorporation in DNA strands, which induces stalled replication forks and thereby replication stress [76]. This would lead to DNA strand breaks after cell division. One hypothesis to explain the negative outcome of 53BP1 assay with this substance could be that 24 h of exposure, as used in our experiments, would be too short to reveal such damage when cells undergo replication stress. It concurs with the absence of genotoxicity via γ-H2AX assay observed by others upon exposure of several cell lines to AZT [77]. Regarding ENU, only one of the tested concentrations led to an increase of 53BP1 foci count, which was a cytotoxic concentration leading to 30% of cell death. Therefore, in the 53BP1 assay, the range of concentrations causing DNA damage for this substance is narrow, either because it may be efficiently repaired at non cytotoxic concentrations, or because the induced DNA lesion is not efficiently detected in the 53BP1 assay, unless at cytotoxic concentrations where the detected strand breaks may originate from DNA fragmentation in deadly cells. ENU causes O^6^AlkG alkylation of DNA, which is repaired by O^6^-methylguanine DNA methyltransferase (MGMT), and which is highly mutagenic but not clastogenic [78]. The 53BP1 assay logically detects DNA double strand breaks but not mutations, which supports this hypothesis.

X-ray irradiation at the dose of 1 Gy led to significant DNA damage as detected in the 53BP1 assay, with a maximum response at 30 min post-irradiation, followed by progressive decrease of 53BP1 foci count (Fig 3(j)), suggesting that double strand breaks (DSB) were progressively repaired, as already reported [29]. Löbrich et al. report that DNA repair kinetics depends on the tested cell line, with some cells having readily repaired the DSB generated by X-rays after 15 min while, in other cell lines, 4 to 8 h are necessary for their effective repair [29]. In HCT116, the repair kinetics is within the same order of magnitude, with 1.5–2 h necessary to repair most of the DSB. Moreover, a number of foci are left unrepaired, with 3.1 ± 1.4 foci remaining 24 h after X-ray irradiation while control cells show 1.3 ± 0.4 foci. These foci remaining 24 h post-irradiation are described to being related to several cellular processes, as discussed by Rothkamm et al. [16]. HCT116 cells are p53-competent but still it is a cancerous cell line, and cancer cell lines are generally radioresistant, therefore such remaining foci were expected.

When comparing the number of foci detected via the 53BP1 assay with that observed via the γ-H2AX assay (Fig 3(k)), i.e., 8 and 6, respectively, the 53BP1 gives a higher number of foci. This is unexpected, since γ-H2AX is reported to form foci on DNA DSBs but also following other cellular events as described in the introduction section, while 53BP1 would be more specific of DNA DSBs. Therefore, one would expect to detect more γ-H2AX foci, compared to 53BP1 foci. Still, we rather believe that this difference is due to a more precise counting of 53BP1 foci by the image analysis system, compared to γ-H2AX foci. Indeed, the images captured after both immunostainings show that the background staining is higher in γ-H2AX images, and that the 53BP1 foci are bigger and brighter than the γ-H2AX foci (S3 Fig in S1 File). Therefore, the image analysis software probably counts more accurately 53BP1 foci, compared to γ-H2AX foci.

Finally, Löbrich et al. observe 15 γ-H2AX foci per cell nucleus at 15 min post-exposure to 1 Gy of X-rays [29]. Here, we observe only 6 γ-H2AX foci per cell nucleus (Fig 3(k)), which is approximately half the number of γ-H2AX foci foci reported in the study by Löbrich et al. It would suggest that in our experimental conditions we do not detect all DNA repair foci, or that the automated microscope used in the present study, which is not a confocal microscope, cannot integrate all the foci in a single image capture. Note also that Löbrich et al. report the foci number 15 min after irradiation, while we report here the foci number 30 min after irradiation. Given the repair kinetics that we observe (Fig 3(j)), it is possible that some DSB have already been repaired 30 min post-irradiation, and that more foci would have been detected 15 min post-irradiation. Another hypothesis is related to the dishes or plates used during the experiment, on which cells are irradiated. We irradiated cells in plastic 96-well plates while Löbrich et al. irradiated cells on glass slides. It has been reported that cells irradiated on glass slides receive direct radiation but also secondary radiation originating from the irradiated glass surface on which cells are grown [79]. The number of γ-H2AX foci has been reported to be twofold greater in cells irradiated on glass slides compared to cells irradiated on plastic material [79], (see [79] for explanations of the physical phenomenon). We observe approximately half the number of γ-H2AX foci in the present study where we used a plastic support, compared to the study of Löbrich et al. where a glass support was used. Consequently, the disparity between the number of foci detected in the study by Löbrich et al. and in the present study is probably due to the material on which cells are irradiated, as well as to the distinct sensitivities and DNA repair capacities of the used cell lines.

### 3.2. Genotoxicity screening of a panel of NBMs, using the 53BP1 assay

#### 3.2.1. Selection of the tested NMs and NBMs, assessment of their impact on cell viability

Having validated the accuracy of the 53BP1 assay on the HCT116 cell line using known chemical genotoxicants as well as X-ray irradiation, we then applied the assay to a series of NBMs having different composition, sizes and functional properties. The NBMs were selected within three categories of NBMs, i.e., metals/metal oxides, organic and mineral NBMs, including silver nanoparticles (AgNP), silver nanoparticles coated with hydroxyethylcellulose (AgHEC), gold nanoparticles (AuNP), gold nanorods (AuNR), carbon nanoparticles (CNP), multi-walled carbon nanotubes (MWCNT), solid lipid nanoparticles (SLN), titanium dioxide (TiO2), magnetite (Fe3O4), hydroxyapatite (HA), hydroxyapatite scaffold (HAsc) and iron-doped hydroxyapatite (FeHA). These materials are described in the Supplementary Information section. Their average size (Z-average, polydispersity index -PdI-) and zeta potential are summarized in S2 Table in S1 File and their size distributions are reported in S4 Fig in S1 File, except for MWCNT that could not be characterized using dynamic light scattering (DLS) as it is not adapted to non-spherical particles, because the contribution of their rotational diffusion is not taken into account by the method [80].

**Fig 4:**
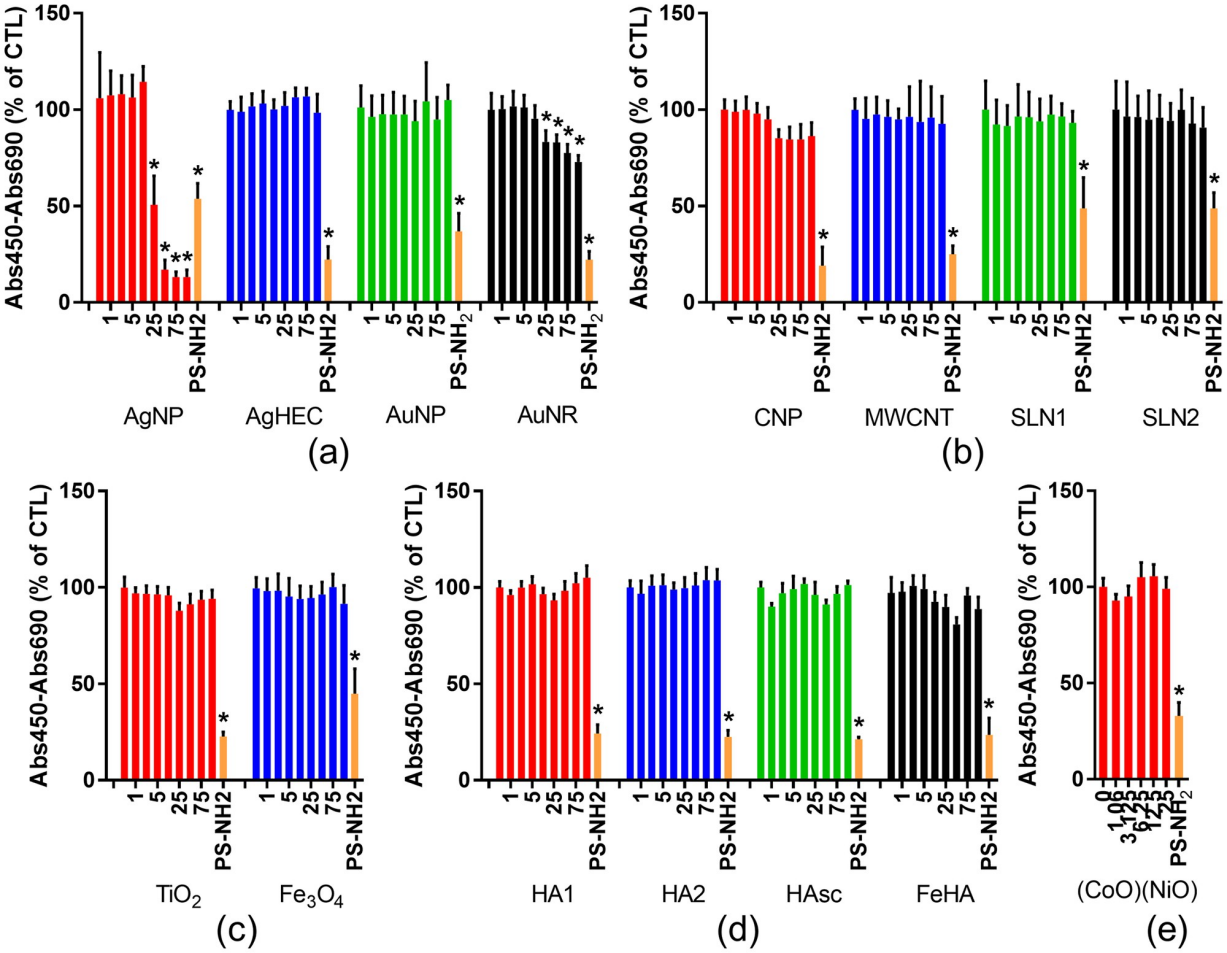
Impact of NBMs on cell viability. Cell viability was evaluated using the WST-1 assay in HCT116 cells exposed to NMs and NBMs. (a) Metal-based NMs and NBMs: silver nanoparticles (AgNP), silver nanoparticles coated with hydroxyethylcellulose (AgHEC), gold nanoparticles (AuNP), gold nanorods (AuNR). (b) Organic NMs and NBMs: carbon nanoparticles (CNP), multi-walled carbon nanotubes (MWCNT), solid lipid nanoparticles (SLN). (c) Metal oxide NMs and NBMs: titanium dioxide (TiO2) and magnetite (Fe3O4). (d) Mineral NMs and NBMs: hydroxyapatite (HA), hydroxyapatite scaffold (HAsc) and iron-doped hydroxyapatite (FeHA). The impact of (CoO)(NiO) on cell viabiliy is also reported, as it used as positive control in the 53BP1 assay. Concentrations of NBMs are expressed in μg/mL (x-axis). Amine-functionalized polystyrene NPs (PS-NH2, 50 nm, 100 μg/mL, 24 h) were used as positive control in each assay (PS-NH2). Depicted are the mean values ± standard deviation of 3 independent experiments with 5 replicates per experiment (n = 15). Statistical significance: *p<0.05, exposed versus control.

As for soluble genotoxins, the impact of these NBMs on cell viability was assessed using the WST-1 assay (Fig 4). When the concentration of NMs exceeds 100–200 μg/mL, NMs may interfere with some toxicity assays, either optically or enzymatically [4]. Although the WST-1 assay is less prone to NM interference than other cytotoxicity assays because NMs are removed from the test medium before final absorption measurement, it is recommended not to exceed 100–200 μg/mL when assessing NM toxicity [58, 71]. In this range of concentrations, most of the tested NBMs exhibited no significant impact on cell viability, except AgNPs and AuNRs. AgNPs caused 50% of cell dehydrogenase activity loss at 25 μg/mL and more than 80% of cell dehydrogenase activity loss at higher concentrations. AuNRs caused 15 to 27% of cell dehydrogenase activity loss when exposed at concentrations ranging from 25 μg/mL to 100 μg/mL. The AgNPs (NM300K, obtained from the nanomaterial repository of the European Joint Research Center (JRC, Ispra, Italy)) have been studied in many previous publications and their cytotoxicity potential is well-documented [81–83].

#### 3.2.2. Response of NMs and NBMs in the 53BP1 assay

**Fig 5:**
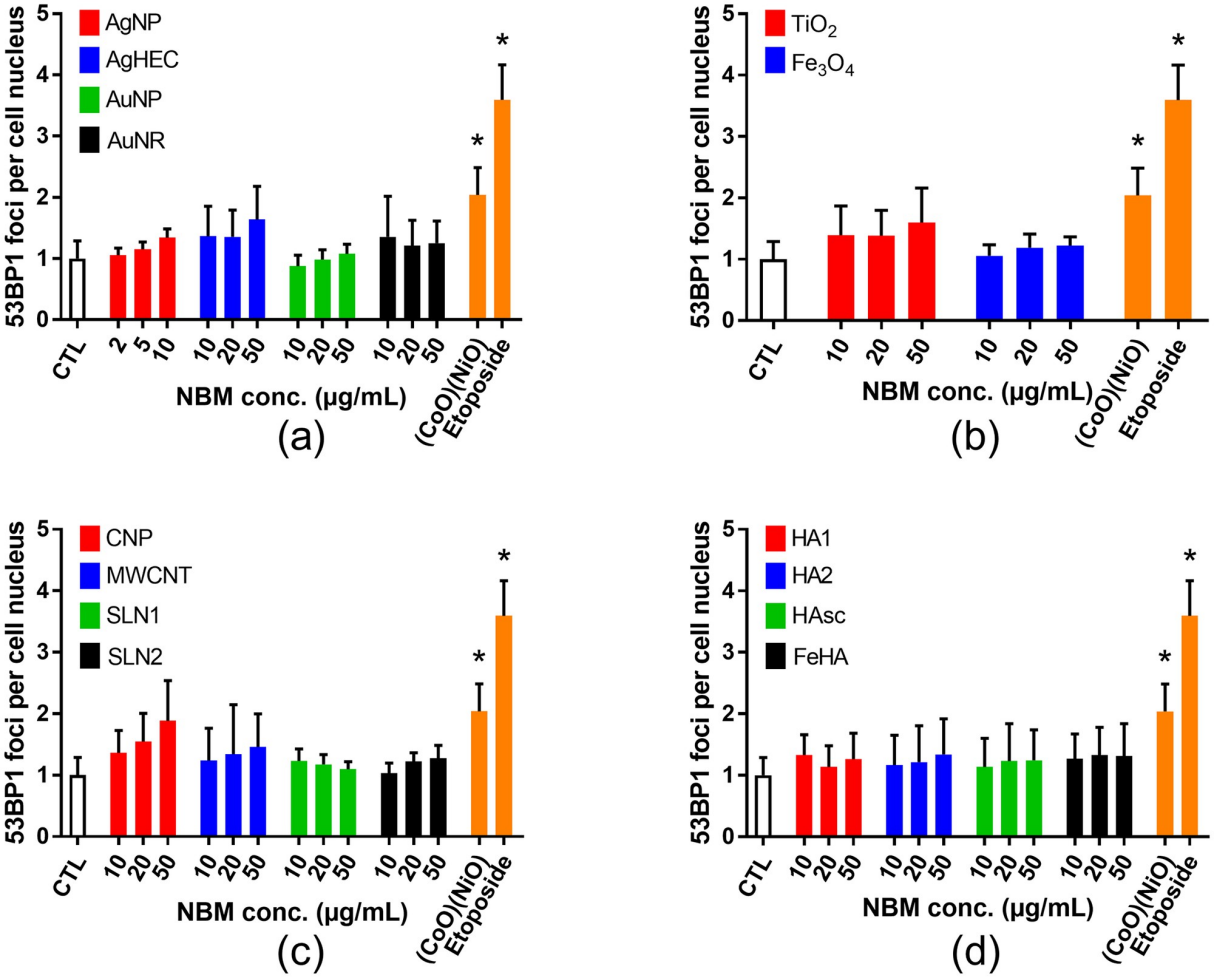
Genotoxicity screening of NBMs using the 53BP1 assay. Genotoxicity was assessed in HCT116 cells exposed to NBMs for 24 h. (a) Metal-based NMs and NBMs; (b) metal oxide NMs and NBMs; (c) organic NMs and NBMs; (d) mineral NMs and NBMs. Cobalt nickel oxide (CoO)(NiO) NPs (20 μg/mL, 24 h) and etoposide (50 μM, 24 h) were included as positive controls. Depicted are the mean values ± standard deviation of 3 independent experiments with 5 replicates per experiment (n = 15). Statistical significance: *p<0.05, exposed versus control.

In order to assess the genotoxicity of all NBMs at comparable concentrations, we chose 50 μg/mL as the highest tested concentration. At this concentration, none of the tested NBMs caused more than 15% of loss of cell viability except AgNPs, which was considered separately due to its much stronger cytotoxic impact. Following the same strategy as the one used for molecular genotoxic agents, three concentrations of NBMs were tested, i.e., 50 μg/mL, 25 μg/mL and 10 μg/mL in order to identify any increase of 53BP1 counts that could be related to increased NBM exposure concentrations. AgNPs were tested at the highest concentration that did not induce any reduction of cell viability as estimated via WST-1 assay, then half and one fifth of this concentration, i.e., 10, 5 and 2 μg/mL (Fig 5).

None of the tested NBMs induced any significant elevation of the 53BP1 foci count, while it significantly increased in both positive controls, i.e., cells exposed for 24 h to 50 μM etoposide or to 20 μg/mL cobalt nickel oxide ((CoO)(NiO)) nanoparticles (Fig 5). The surfactants used for AgNPs, SLN1 and SLN2, Fe3O4 and AgHEC were also tested using the 53BP1 assay; they did not induce any elevation of 53BP1 foci count (S5 Fig in S1 File). Typical images obtained in these 53BP1 assays are shown in S6 Fig in S1 File.

Here, (CoO)(NiO) was used as positive control owing to the co-existence of cobalt oxide and nickel oxide in this substance, both having been described as being genotoxic (see, for instance, [84–86]). Moreover, we confirmed that the assay could capture genotoxic events caused by a NM with acknowledged genotoxicity by assessing the response to ZnO NM110 NM (from the JRC repository), which have been previously reported to be genotoxic [87]. With this NM, we observed a progressive increase of 53BP1 foci counts as ZnO concentration increased (S7 Fig in S1 File). Therefore, this assay can positively capture the genotoxicity of some acknowledge genotoxic NMs.

The genotoxicity of the AgNPs tested here (NM300K) has already been evaluated in several cell systems, especially in lung cells and intestinal cells, either grown as monoculture or co-culture or triple culture. NM300K is reported to be genotoxic in some studies, in particular when genotoxicity is assessed using the comet assay [81, 88, 89]. It is reported to either positively or negatively respond in the micronucleus assay (see [81–83, 90], respectively), although the cell systems and applied concentration differ in these studies, with some very low concentration tested in one study compared to the others [90]. Moreover, in one of these studies its cytotoxicity is not evaluated before genotoxicity assessment, therefore the observed DNA damage could be due to fragmented DNA in dead cells [82]. Therefore, no clear consensus can be found from these previous studies regarding the potential genotoxicity of this substance. Interestingly, Bobyk et al. tested NM300K genotoxicity both in the comet assay, micronucleus assay and 53BP1 assay on A549 cells, and obtained a positive result in the alkaline comet assay, but not in the Fpg-modified comet assay, nor in the micronucleus and 53BP1 assays. These assays do not detect the same type of DNA lesion, the comet assay detects strand breaks and alkali-labile sites, as well as Fpg-sensitive sites such as 8-oxo-dGuo in its Fpg-modified version, the micronucleus assay detects chromosomal breaks or losses and the 53BP1 assay detects double strand breaks. Therefore, one may recommend to systematically comparing several assays in order to have a clear view on the DNA damaging potential of NBMs, but also to choose the test system appropriately, as the DNA damaging potential, due to different sensitivity and different DNA damage response, may vary across several cell types.

#### 3.2.3. Genotoxicity NMs and NBMs as assessed via the micronucleus (MN) assay

**Fig 6:**
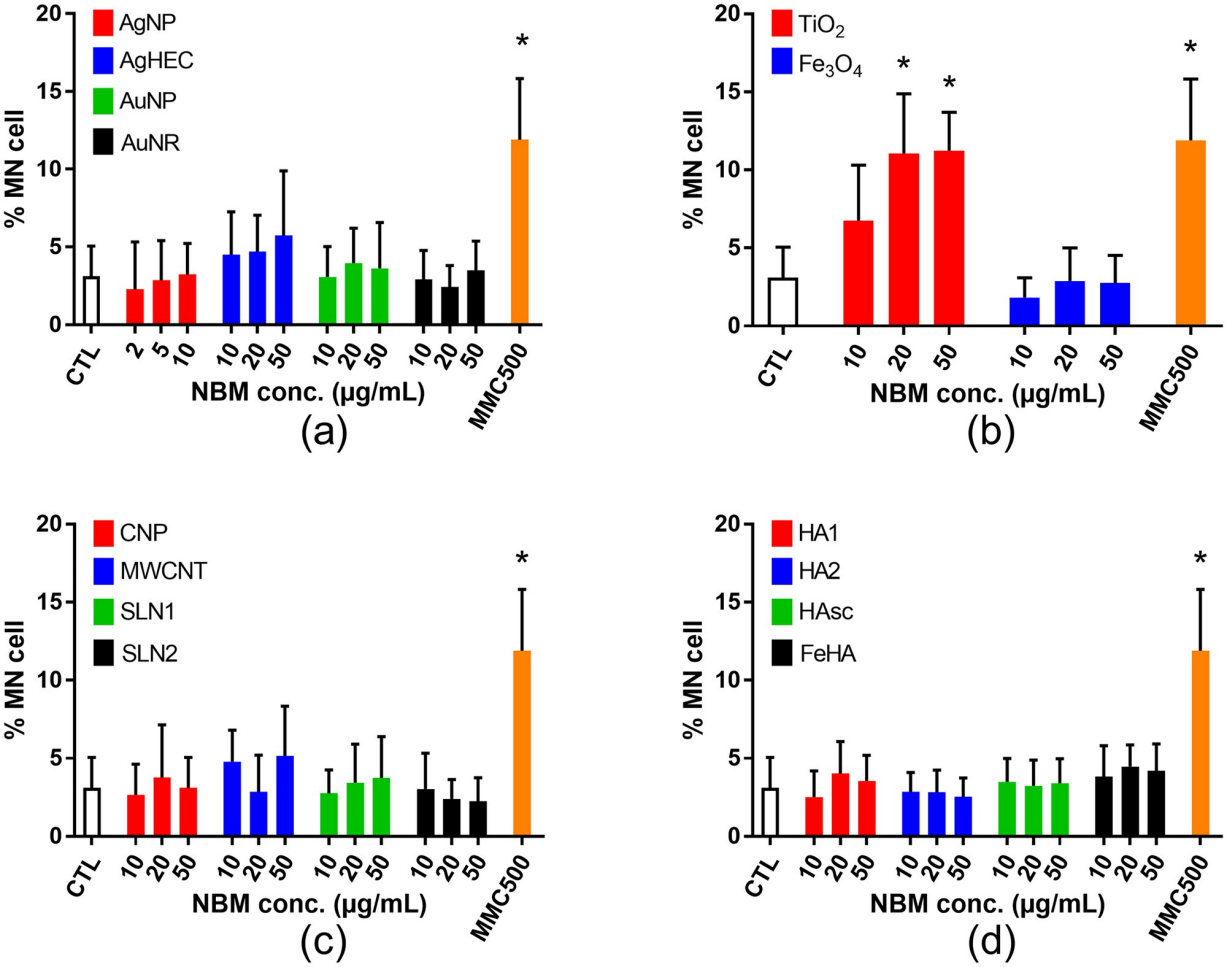
Genotoxicity screening of NBMs using the cytochalasin-blocked micronucleus assay. The per-centage of micronucleated cells in the binucleated cell population after 24 h exposure to the se-lected NBMs is shown. (a) Metal-based NMs and NBMs; (b) metal oxide NMs and NBMs; (c) organic NMs and NBMs; (d) mineral NMs and NBMs. Mitomycin C (MMC500, 500 ng/mL, 24 h) was used as a positive control. Depicted are the mean values ± standard deviation of 3 independent experiments with 5 replicates per experiment (n = 15). Statistical significance: *p<0.05, exposed versus control.

To confirm the non-genotoxicity of the tested NBMs, we applied the cytokinesis-blocked micronucleus assay in the same exposure conditions (Fig 6). None of the tested NBMs induced any elevation of the micronucleus count except TiO_2_ NPs, which increased the micronucleus count up to fivefold compared to unexposed cells, similar to the positive control (mitomycin c, MMC).

A recent review reports that the in vitro MN assay applied to a variety of NMs, i.e., metal oxides (Al-, Cu-, Ce-, Fe-, Si-, Ti-, Y-, and Zn-oxides), metals (Au, Ag), carbon based (fullerenes, SWCNTs, MWCNTs) and some combination materials (QDs, WC-Co) rarely results in increased MN frequency [9]. Positive outcome in the cytokinesis-blocked micronucleus assay has been observed in cells exposed to single- and multi-walled carbon nanotubes [91–93], Au- and Ag-NPs [94, 95], SiO_2_ nanoparticles, both amorphous and crystalline [96–98], magnetite [99, 100], CeO_2_ [101] and TiO_2_ nanoparticles [102–104], although the test system differs in these studies. Our present results, obtained via the micronucleus assay, confirm the potency of TiO_2_ NPs to induce genotoxic damage, while the Au NP, Ag NP and MWCNT tested here did not lead to a positive response in this assay. Regarding TiO2-NPs, the three studies reporting positive outcome in the MN assay have been performed with P25 Evonik particles, which are mixed phase anatase/rutile NPs and with 70 nm anatase NPs. Moreover, E171 TiO2 particles (~120 nm in diameter) cause a significant induction of micronuclei in HCT116 at 50–500 μg/mL [105]. The TiO2 NPs used in the present study, i.e., NM101 from the JRC nanomaterial repository, is a pure anatase TiO2 NP with primary diameter ~5 nm [106], aggregated as >300 nm clusters. Therefore, physico-chemical characteristics of all these TiO_2_ particles are very different, which makes it difficult to compare the outcomes of genotoxicity assays. TiO2 NPs did not increase the number of 53BP1 foci, while they induced a positive response in the micronucleus assay. The most probable hypothesis to explain this discrepancy is, here again, that the 53BP1 and the micronucleus assay do not monitor the same type of DNA damage. 53BP1 specifically detects DNA DSBs, which may be caused by exogenous agents such as ionizing radiation, some chemicals, anti-cancer drugs or some environmental stress, but also by endogenous cellular processes such as apoptosis, replication fork collapse during the replication of damaged DNA or some DNA repair processes [16]. Conversely, micronuclei result from chromosomal damage, both chromosome breakage, loss or rearrangements [107], which originate from unrepaired strand breaks, but also from malsegregation of chromosomes during the mitosis due to defects in the mitotic spindle or centromere, or from default in chromosome condensation before the metaphase. Therefore, some DNA damage detected via the micronucleus assay would be also detected via the 53BP1 assay, but not all of them. Especially chromosome malsegregation, which have been shown to be caused by some TiO_2_-NPs (see [108], who show that chronic exposure of NIH 3T3 cells to 15 nm TiO_2_ NPs alters chromosome alignement and segregation during anaphase and telophase, together with aberrant chromosome segregation, and as a consequence increased micronucleus number), would lead to an increase in the micronucleus number, but not in the 53BP1 foci number. This underlines the necessity to use several complementary genotoxicity assays in order to properly characterise the genotoxic potential of a substance, as none of these assays can detect the whole range of possible genotoxic events. Moreover, some micronuclei may occur in cells undergoing apoptosis [109]. Apoptosis has not been tested in the present study, but TiO_2_ NPs have been reported as potent apoptosis inducers, especially in HCT116 cells at high concentration (125 μg/mL) [73]. In the present study, when observing the nuclei of cells exposed to TiO2 NMs, some nuclei appear to show more intense staining with Hoechst 33342 (S6 Fig in S1 File), which may reflect chromatin condensation in the early stages of apoptosis. Therefore, although it may necessitate further investigation, early apoptosis may also explain why some micronuclei are observed in HCT116 cells exposed to TiO_2_ NMs, while no positive response is observed in the 53BP1 assay.

Regarding the NBMs showing negative outcome in the MN assay in the present study but positive outcomes in other studies, the following assumptions could be made. First, the MWCNT tested here are entangled and thin (11–16 nm) [110, 111], compared to those carbon nanotubes that were found to be genotoxic by others [91–93]. MWCNT toxicity, especially genotoxicity, is known to increase with diameter [112]. The genotoxicity of this benchmark material has already been reported in several studies, and found to be less genotoxic than other carbon nanotubes [113]. Regarding CNPs, they could be considered less toxic than carbon nanotubes since carbon nanotube toxicity is related to their high aspect ratio [114]. In the case of AgNPs, the Ag-NPs showing significant genotoxicity in the study by Li et al. are smaller than those tested here (5 nm vs. 15 nm), and they show only a weak positive outcome in the MN assay (1.6% increase compared to control) [100]. Moreover, they were tested at concentrations leading to significant decrease of relative population doubling (60% and 40% of the population doubling in unexposed cells), while here we tested their genotoxicity at concentration that did not induce any significant reduction of cell viability. We previously used the 53BP1 and MN assays to evaluate the genotoxicity of several Ag-NPs on A549 cells, including the same Ag NP as in the present study (i.e., NM300K from the JRC nanomaterial repository), and we found no significant MN induction whatever the NP size and coating on this cell line [81], which confirms the present result. Finally, the magnetite particles tested here are coated with polyethylene glycol and poly(lactic-co-glycolic acid) (PEG-PLGA) and Singh et al. reported that the coating of iron oxide nanoparticles leads to the loss of their DNA damaging potential [115]. This would explain why we do not observe any genotoxicity with the Fe3O4-PEG-PLGA NBMs.

### 3.3. Screening for cellular oxidative stress induced by NBMs

**Fig 7:**
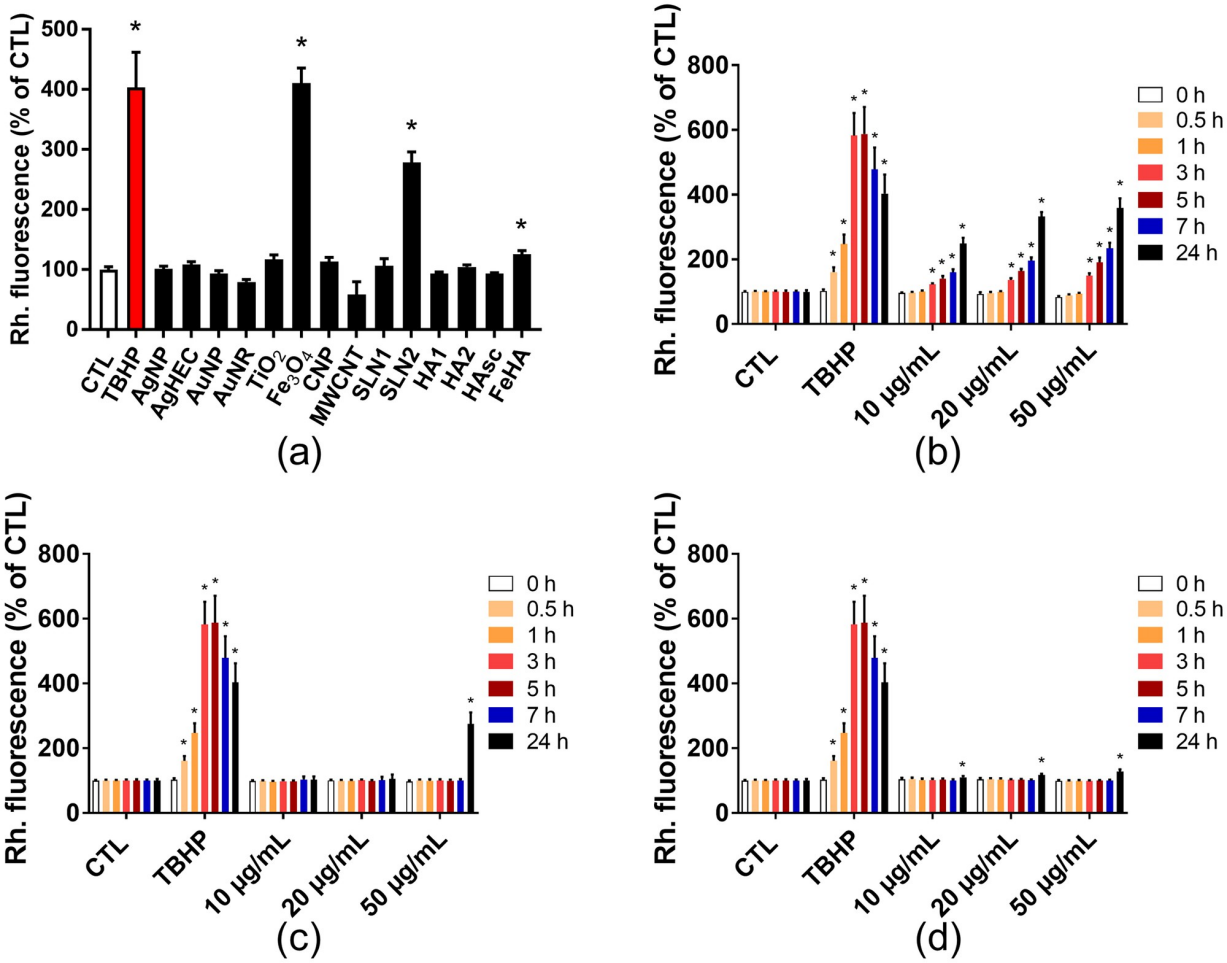
ROS levels in cells exposed to NBMs. ROS levels were assessed using the DHR123 assay, in cells exposed for 24 h to the whole series of tested NMs and NBMs (a), or for 0.5–24 h to Fe3O4 (b), SLN2 (c) or FeHA NPs (d). Tert-butyl hydroperoxide (THBP, 250 μM) was used as positive control. Depicted are the mean values ± standard deviation of 3 independent experiments with 5 replicates per experiment (n = 15). Statistical significance: *p<0.05, exposed versus control.

Finally, since NBM genotoxicity has been related to their propensity to trigger oxidative stress [12], the ROS content was measured in HCT116 cells after exposure to the series of NBMs tested for their genotoxicity. Among all the tested NBMs, only Fe3O4, SLN2 and FeHA triggered a significant elevation of the intracellular ROS content (Fig 7(a)). A concentration-dependent increase of intracellular ROS content was observed in HCT116 cells exposed to Fe3O4 particles (Fig 7(b)), while SLN2 (Fig 7(c)) and FeHA (Fig 7(d)) only triggered ROS content elevation after 24 h of exposure, and only at 50 μg/mL for SLN2 (Fig 7(c)).

Only TiO2 NPs induced genotoxic stress to HCT116 cells, and only in the MN assay, without causing significant elevation of intracellular ROS content, as measured with the dihydrorhodamine 1,2,3 (DHR123) probe. Conversely, Fe3O4 NPs caused significant and time-dependent elevation of ROS content without showing any genotoxicity as revealed by both the 53BP1 and MN assays. This proves that although NM genotoxicity might be related to oxidative stress, as generally accepted, other mechanisms can also explain their DNA damaging potential. Regarding TiO2 NPs, their ability to interact with the mitotic spindle and impair chromosome segregation during mitosis has been reported [108], as well as their ability to impair the cell’s DNA repair capacity [116, 117], which could lead to DNA damage without causing any ROS elevation. Still, increased ROS content in cells exposed to TiO2 NPs has been widely demonstrated (for review, see [118]). TiO2 NPs produce both hydroxyl radical (OH°) in presence of H2O2 and singlet oxygen. OH° is the most potent radical to attack the DNA backbone [119], but its reactivity is so high that it does not diffuse into cells. Therefore, if produced in the cytoplasm in the vicinity of accumulated TiO2 NPs, it may not reach the DNA and cause strand breaks. Conversely, singlet oxygen, if formed in the cytoplasm, could be transferred to the cell nucleus, as its lifetime is much longer, and could damage DNA [120]. Using H2-DCF-DA assay, we previously showed ROS induction by a series of TiO2 NPs varying in their crystal structure, shape and size, in A549 cells [117]. One hypothesis to explain such discrepant results could be that DHR123 assay may not be sensitive enough to detect ROS generated by the particular TiO_2_ NP used in the present work, especially singlet oxygen. It would be interesting to quantify and compare the sensitivity of both DHR123 and H2DCFDA assays, which would be the topic of a future study. Still, the DHR123 and H2-DCF-DA probes have the same reaction mechanism, it is more probable that the discrepant results originate from the different sensitivities of the used cell lines, as well as the different physico-chemical characteristics of the assessed TiO2 NPs. Other measurement methods, based on flow cytometry, have also been reported to be more sensitive in ROS detection than plate spectrofluorometric measurement as used in the present study (N. Ruijter, RIVM, personal communication). Although flow cytometry is not as high throughput and not as classically used as spectrofluorometry, it may make possible the detection of low-grade elevation of ROS levels that could explain the genotoxic damage caused by these TiO2 NPs. In addition, a recent study published by the BIORIMA consortium and performed with the same TiO2 NPs (NM101), using the U2OS- nuclear factor (erythroid-derived 2)-like 2 (NRF2) reporter cell line, did not show any activation of the NRF2 pathway [121], which is usually activated when cells undergo significant oxidative stress. Therefore, TiO2 NMs might generate some reactive species that are not reactive towards the DHR123 probe, or at levels that are too low to be detected by this assay, and might not generate sufficient oxidative stress to trigger the NRF2 pathway but might travel to the cell nucleus and damage DNA. Interestingly, this U2OS-NRF2 system showed positive, although limited, response of the same Fe3O4 NPs as those used in the present study, which is consistent with the current observation. It is also consistent with some in vitro and in vivo studies showing oxidative stress being triggered by Fe3O4 NPs (for instance, see [122–125] in vivo and [126–130] in vitro), via a mechanism based on the Fenton reaction as discussed by Burello et al. [131]. In this reaction, hydrogen peroxide originating from cellular processes oxidizes Fe2+ from Fe3O4 (magnetite) NMs to Fe3+, forming Fe2O3 (maghemite) clusters. Again, within this reaction that may occur inside cells, H2O2 would be simultaneously decomposed into OH^-^ and OH° while oxidizing Fe3O4, and these species cannot diffuse into cells and therefore cannot reach the nucleus and attack DNA, explaining that no DNA damage is observed for Fe3O4 NPs via the 53BP1 assay. The PEG-PLGA coating on Fe3O4 NPs is supposed to avoid their DNA damaging potential [115], via reducing their surface reactivity, but we previously showed that this coating does not totally cover the surface of the Fe3O4 particles tested here [44]. This might explain why they show a significant ROS generation potential, without causing any DNA damage. Alternatively, the reactive oxygen species resuting from Fe3O4 impact on HCT116 cells might cause a type of DNA damage that is not detected by the 53BP1 assay. Indeed, the 53BP1 assay detects DSBs, while ROS would rather induce DNA base oxidation that are easily repairable by the base excision repair DNA repair process.

Regarding FeHA particles, the observed ROS can originate from the very small amount (~ 2 wt%) of maghemite (Fe2O3) nano-nuclei exposed at their surface, as identified earlier [50], which would generate ROS.

Taken together, these results highlight two distinct behaviors of metal oxide NMs, TiO2 triggering DNA damage without showing any significant elevation of the intracellular ROS level, although this would need to be confirmed via more sensitive methods, and Fe3O4 drastically increasing the intracellular ROS level without causing any DNA double strand breaks or chromosomal damage. More experiment would be needed to prove that no other DNA damage is caused by Fe3O4 particles, such as the quantification of oxidized DNA bases.

## 4. Conclusions

A new method for nano(bio)material genotoxicity evaluation is proposed, based on the labelling and counting of foci of the 53BP1 DNA repair protein within the nucleus of exposed cells. Foci are counted using a high throughput screening/high content analysis automated fluorescence imaging and analysis system. The performance of this assay was confirmed through the use of a series of reference genotoxic substances, proposed by the ECVAM to be tested when developing new genotoxicity assays. Then, the assay was applied to nano(bio)materials of varying compositions, which were also tested via the micronucleus assay. All reference genotoxic substances produced the expected outcome, while none of the tested nano(bio)materials showed any significant elevation of 53BP1 foci count, except the positive control, which was (CoO)(NiO), and ZnO NM110 (JRC) nanoparticles, which are acknowledged in the literature as being genotoxic. In the micronucleus assay, TiO2 nanoparticles resulted in a positive outcome, which can be explained by their ability to impair chromosome segregation during the mitosis rather than by a DNA breaking potential, and therefore would logically not be detected by the 53BP1 assay. This new assay is sensitive, cost-effective, robust and easy to implement, which makes it a relevant assay for high throughput genotoxicity screening in the frame of New Approaches and Methodologies development. In this study, the 53BP1 assay has been developed on the HCT116 human epithelial intestinal cell line; it would now need to be reproduced in a cell line that has been validated for the evaluation of genotoxicity in a regulatory context, such as those recommended in OECD guidelines, and/or on a cell line that expresses relevant amounts of Phase I and Phase II metabolic enzymes, transporters and nuclear receptor so that it can capture the genotoxicity of byproducts resulting from the metabolization of some genotoxins.

## Supporting information

S1 File Nanomaterial genotoxicity evaluation using the high-throughput p53-binding protein 1 (53BP1) assay. (DOCX)

S1 Graphical abstract (PNG)
