# Supplementary material for: Nanomaterial genotoxicity evaluation using the high-throughput p53-binding protein 1 (53BP1) assay
Source: PLoS One. 2023 Sep 15;18(9):e0288737. doi: 10.1371/journal.pone.0288737 (PMC10503773; doi:10.1371/journal.pone.0288737)
Supplement: S1 File — (DOCX) [file pone.0288737.s001.docx]

Supporting information for: Nanomaterial genotoxicity evaluation using the high-throughput p53-binding protein 1 (53BP1) assay

Maelle Fontaine^1^, Eline Bartolami^1^, Marion Prono^1^, David Béal^1^, Magda Blosi^2^, Anna L. Costa^2^, Costanza Ravagli^3^, Giovanni Baldi^3^, Simone Sprio^2^, Anna Tampieri^2^, Ivana Fenoglio^4^, Lang Tran^5^, Bengt Fadeel^6^, and Marie Carriere^1,*^

^1^Univ. Grenoble Alpes, CEA, CNRS, IRIG, SyMMES‑CIBEST, 38000 Grenoble, France; maelle.fontaine@cea.fr, eline.bartolami@cea.fr, marion.prono@cea.fr, david.beal@cea.fr, marie.carriere@cea.fr

^2^National Research Council, Institute of Science, Technology and Sustainability for Ceramic Materials ISSMC-CNR (former ISTEC-CNR), Via Granarolo 64, 48018 Faenza, Italy; magda.blosi@istec.cnr.it, simone.sprio@istec.cnr.it, anna.costa@istec.cnr.it

^3^Ce.Ri.Col, Colorobbia Consulting S.R.L, 50059 Sovigliana-Vinci, FI, Italy; ravaglic@colorobbia.it

^4^Department of Chemistry, University of Turin, 10125 Turin, Italy; ivana.fenoglio@unito.it

^5^Institute of Occupational Medicine, Edinburgh, Midlothian EH14 4AP, UK; lang.tran@iom-world.org

^6^Institute of Environmental Medicine, Karolinska Institutet, 171 77 Stockholm, Sweden; bengt.fadeel@ki.se

1. Detailed description of the used NMs and NBMs

1.1. Metal and metal oxide NMs and NBMs

- AgNP: JRC NM300K suspension, based on Ag nanoparticles. Benchmark material. The characterization of this material is described in the Joint Research Center (JRC) reports [1,2].

-AgHEC: Ag nanoparticles coated with hydroxyethyl cellulose (HEC), dispersed in water, diameter ranging from 5 to 20 nm. Applications: superficial coating and additive for anti-bacterial purposes properties. Provided by CNR-ISSMC [3,4].

-Au NPs: gold nanoparticles coated with citrate and stabilized with PVP, with diameter ranging from 5 to 20 nm. Applications: drug delivery, in vivo imaging (X-ray contrast agent), antimicrobial, photodynamic therapy. Provided by Colorobbia [5-8].

-Au NRs: Aqueous suspension of Au nanorods functionalized with 16-mercapto hexadecanoic acid (MHDA) in tris borate (TB) and ethylenediaminetetraacetic acid (EDTA). Mean diameter of 28 x 8 nm. Application: theranostic on cancer cells via hyperthermic treatment by laser irradiation (800-1080 nm), drug delivery, in vivo imaging and contrast agent. Provided by Colorobbia [5-8].

-TiO2 NPs: JRC NM101, anatase, primary diameter 5-7 nm, aggregated. Benchmark material. For detail on physico-chemical characterization, see the JRC report on TiO2 materials [9].

-Fe3O4 PEG-PLGA: magnetite NPs coated with PLGA-b-PEG-COOH block copolymer polymer, biocompatible, diameter in the range of 15 nm. Applications: theranostic purposes as magnetic hyperthermia agent, imaging by MRI (T2 signal contrast agent) and drug delivery via active targeting. Provided by Colorobbia [10,11].

1.3. Organic NMs and NBMs

-MWCNT: JRC NM400, multi-walled carbon nanotube. Benchmark material. For details on physico-chemical characterization, see the JRC report on multi-walled carbon nanotubes [12].

-CNP-PEG: elemental nanoparticles of elemental carbon prepared from a saccharide precursor via hydrothermal carbonization with mean diameter of ~ 70 nm. Applications: carrier for drug delivery, photo-dynamic and photo-thermal therapy of cancer. Provided by University of Torino.

- SLN1: solid lipid nanoparticle in water colloidal suspension loaded with melatonin, with hydrodynamic diameter of 340 nm (PdI 0.4). Applications: food supplements (syrup) as they make possible the water dispersion of hydrophobic drugs. Provided by Nanovector.

-SLN2: solid lipid nanoparticles in water colloidal suspension. Used as excipient for eye drops, obtained by warm microemulsion method. Mean hydrodynamic diameter of 170 nm with a PDI of 0.14. Composed of lecithins, sunflower oil, water (phosphate buffer pH=7). Provided by Nanovector.

1.4. Mineral NMs and NBMs

-HA1: commercial hydroxyapatite nanopowder from Merck SigmaAldrich (#677418), mean diameter around 200 nm. Benchmark material.

-HA2: calcium hydroxyapatite, i.e., synthetically prepared calcium phosphate mineral obtained in the form of powders, possibly containing a fraction of sub-micron particles, and of dispersed nanoparticles (20-200 nm). Applications: bone fillers, bone augmentation (all types), dental implants; nanomedicine: drug delivery systems (hydroxyapatite and amorphous calcium phosphate). Provided by ISSMC-CNR.

- Fe-HA: iron hydroxyapatite obtained as a powder, possibly containing a fraction of sub-micron particles, and of dispersed nanoparticles (20-200 nm). Applications: bone fillers, bone augmentation; nanomedicine: drug delivery system with remote activation, cell therapy, gene therapy. Provided by ISSMC-CNR [13,14].

-HA-Coll-scaffold: hybrid porous scaffold of magnesium-doped hydroxyapatite (MgHA) nucleated in situ of collagen fibers, the fibrous microstructure is very close to the real human bone. Applications: bone augmentation, bone substitution. Provided by Finceramica [15-17].

2. Supplementary tables

Table S1. List of tested genotoxic substances*^a^*

| **ECVAM group** | **Substance** | **Mode of genotoxic action** | **Cmax** | **Tested concentrations** |
| --- | --- | --- | --- | --- |
| Group 1 | Methyl methanesulfonate (MMS) | N7 alkylation | 500 µM | 80, 200, 400 µM |
|  | N-nitroso-N-ethylurea (ENU) | O6 alkylation | 1.25 mM | 0.2, 0.5, 1 mM |
|  | Hydroquinone | Aneugen | 250 µM | 15, 37.5, 75 µM |
|  | Taxol | Aneugen | <62.5 µM | 5, 12.5, 25, 50 µM |
|  | Etoposide | Topoisomerase II inhibitor | <62.5 µM | 10, 25, 50 µM |
|  | Azidothymidine (AZT) | Incorporated into DNA, replication stress | <20 µM | 2, 5, 10, 20 µM |
|  | Aflatoxin B1 (AFB1) | AFB1-FAPy and AFB1-N7-gua DNA adducts | 3 µM | 0.4, 1, 2 µM |
| Group 3 | Di(2ethyl hexyl)phtalate (DEHP) | Non genotoxic ; peroxisome proliferation | <6.25 mM | 0.5, 1.25, 2.5 mM |
|  | Propyl gallate (PG) |  | <125 µM | 20, 50, 100 µM |

*^a^*Cmax: highest concentration that did not induce any reduction of the cell metabolic activity using the WST-1 assay. This concentration was used to calculate appropriate concentrations for genotoxicity testing, i.e., the Cmax, then Cmax/2 and Cmax/5.

Table S2. NBM size distribution and zeta potential*^a^*

| Group | N(B)M | Z-ave. H2O | PdI H2O | Z-ave. medium | PdI medium | Z-ave. medium 24h | PdI medium 24h | z (mV) H2O | z (mV) medium | Endotoxin level (EU/mL) |
| --- | --- | --- | --- | --- | --- | --- | --- | --- | --- | --- |
| Metal/metal oxide | AgNP | 31 | 0.482 | 49 | 0.107 | 38 | 0.319 | -6 | -12 | sterile |
|  | AgHEC | 411 | 0.428 | 197 | 0.273 | 68 | 0.280 | 2.8 | -6 | <1 |
|  | AuNP | 29 | 0.292 | 22 | 0.431 | 32 | 0.419 | -18 | -7 | 100.5 |
|  | AuNR | 32 | 0.626 | 74 | 0.528 | 91 | 0.333 | -36 | -7 | 0.3 |
|  | TiO2 | 320 | 0.427 | 354 | 0.416 | 286 | 0.411 | -21 | -13 | Sterile |
|  | Fe3O4 | 38 | 0.161 | 145 | 0.481 | 126 | 0.424 | -30 | -12 | 2.0 |
| Organic | CNP | 160 | 0.203 | 189 | 0.196 | 162 | 0.162 | -48 | -11 | 0.4 |
|  | SLN1 | 202 | 0.335 | 124 | 0.520 | 103 | 0.575 | -71 | -9 | Interference |
|  | SLN2 | 160 | 0.212 | 111 | 0.549 | 101 | 0.369 | -49 | -9 | 0.5 |
| Mineral | HA1 | 156 | 0.251 | 146 | 0.297 | 138 | 0.258 | -21 | -12 | Sterile |
|  | HA2 | 195 | 0.376 | 160 | 0.404 | 108 | 0.622 | -18 | -13 | <1 |
|  | FeHA | 155 | 0.226 | 148 | 0.320 | 122 | 0.477 | -14 | -13 | interference |

*^a^*NBM Hydrodynamic diameters (Z-average, nm), polydispersity indexes (PdI) and zeta potentials (ζ, mV) were measured using a Malvern zetasizer, NanoZS series, in suspensions of NBM in water (Z-av. water, PdI water and ζ(mV) water) or in exposure medium (Z-av. medium PdI medium and ζ(mV) medium). In exposure medium, they were measured immediately after their dispersion (Z-av. medium; PdI medium) or after 24 h of incubation at 37°C, 5% CO2 (Z-av. medium 24 h; PdI medium 24 h).

3. Supplementary figures

Figure S1. 53BP1 assay for genotoxicity assessment of model genotoxicants. HCT116 cells were exposed to (a) etoposide (Etop), methyl methanesulfonate (MMS), hydroquinone (Hydroq), taxol or to (b) N-nitroso-N-ethylurea (ENU), Di(2ethyl hexyl)phthalate (DEHP), azidothymidine (AZT), propyl gallate (PG), aflatoxin B1 (AFB1) at non cytotoxic and cytotoxic concentrations for 24 h and then fixed, immunostained and analysed for 53BP1 foci using automated fluorescence microscopy. Depicted are the mean number of foci per cell nucleus ± standard deviation of 1 to 3 independent experiments with 5 replicates (wells) per experiment, and 500 cells counted in each of the measured well. Statistical significance: *p<0.05, exposed versus control.

**
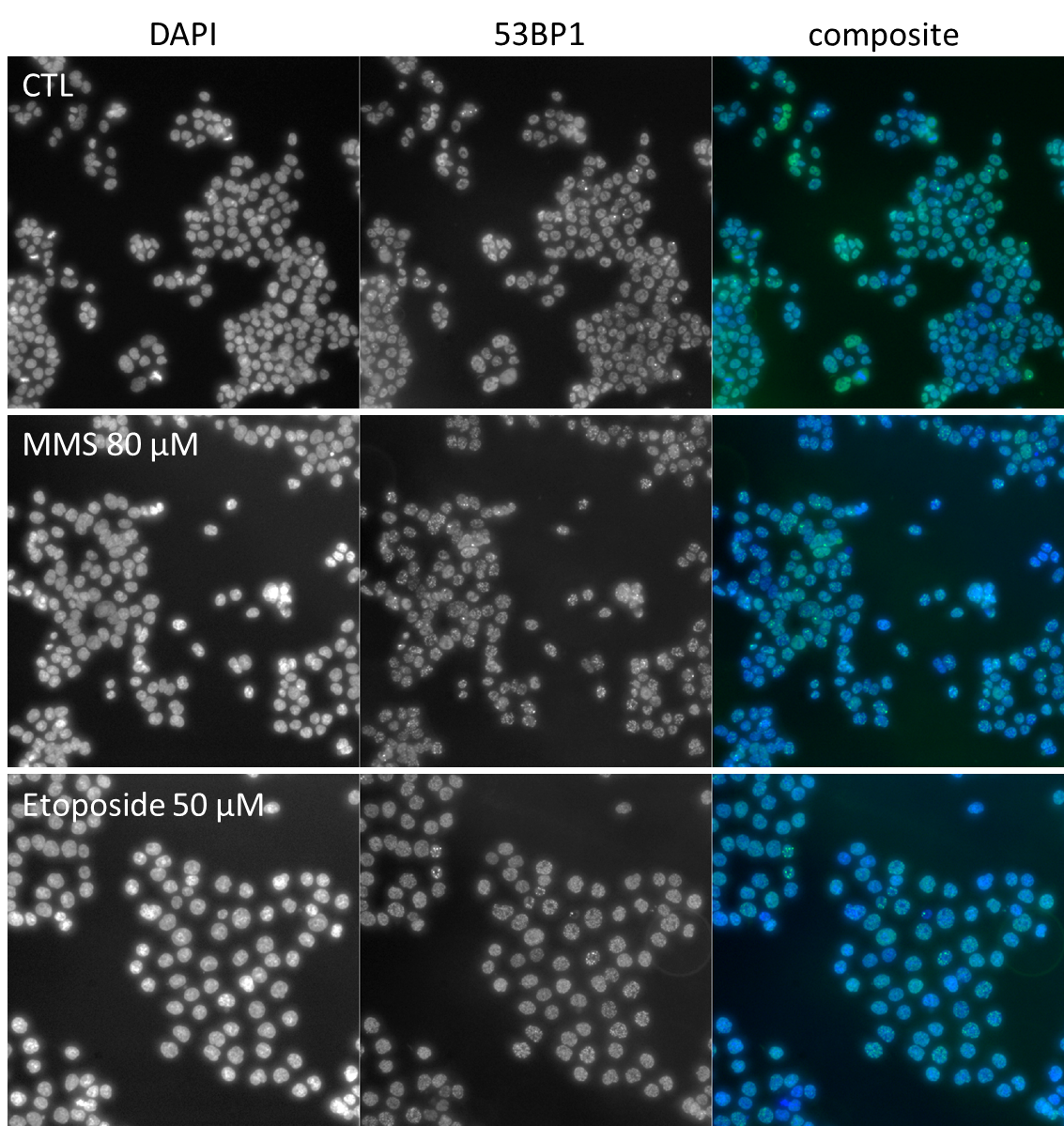
**

Figure S2. Typical images obtained in the 53BP1 assay on reference genotoxic substances. Images were recorded on HCT116 cells exposed for 24 h to 80 µM of MMS or to 50 µM of etoposide, and then immunostained with a 53BP1 antibody (green) and counterstained with Hoechst 33342 (blue, cell nucleus). The composite image is obtained by merging Hoechst 33342 and 53BP1 image, with artificial blue and green colours.


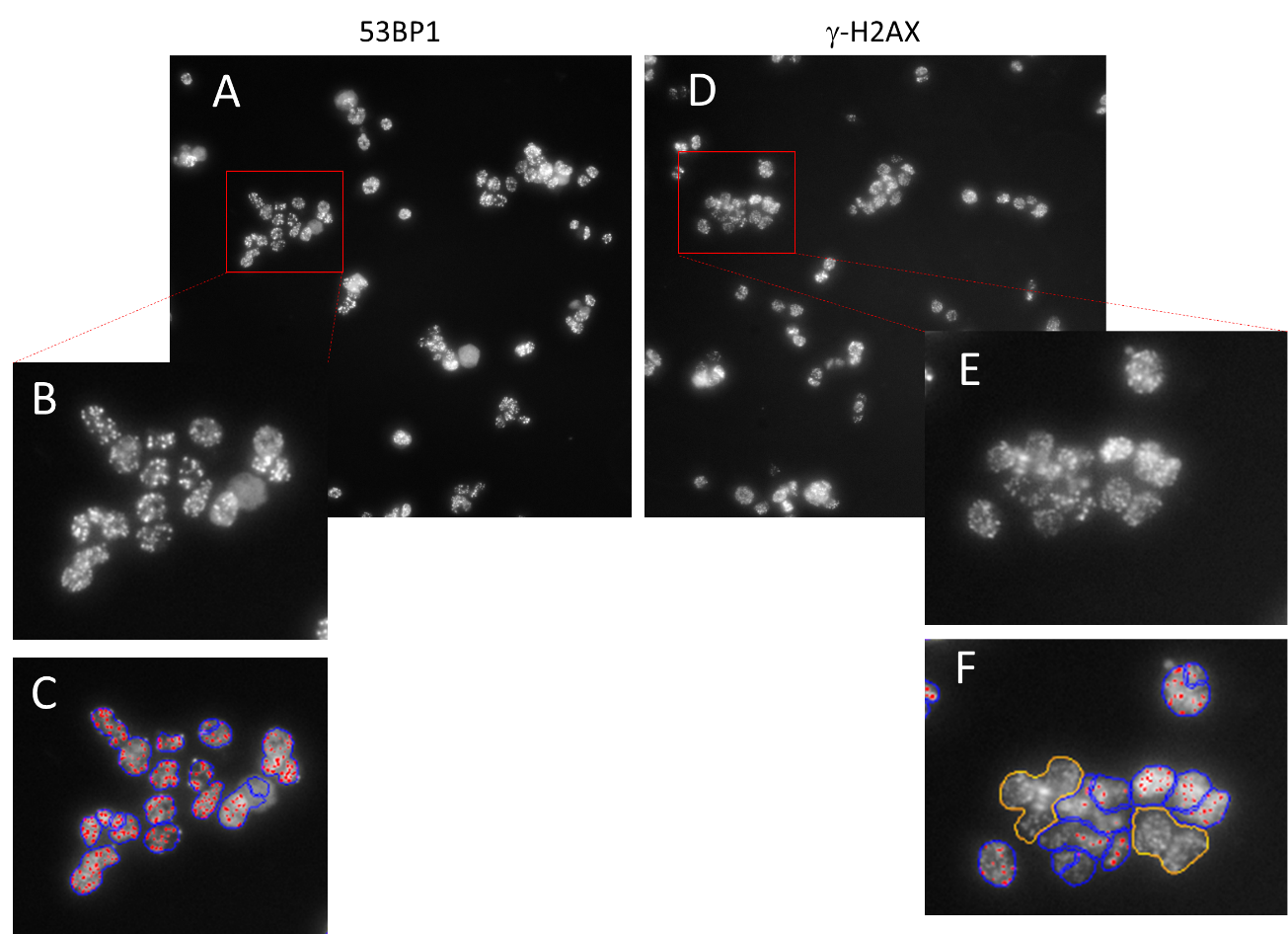


Figure S3. Typical images obtained in the 53BP1 and γ-H2AX assays on cells exposed to 1 Gy irradiation. Images were recorded on HCT116 cells exposed to 1 Gy of X-ray irradiation, then immunostained with an anti-53BP1 antibody (A-C) or an anti-γH2AX antibody (D-F). B and E are higher magnification images of the area delineated with a red square in A and D, respectively. C and F are the results of image segmentation by HCS Studio of B and E areas, respectively.


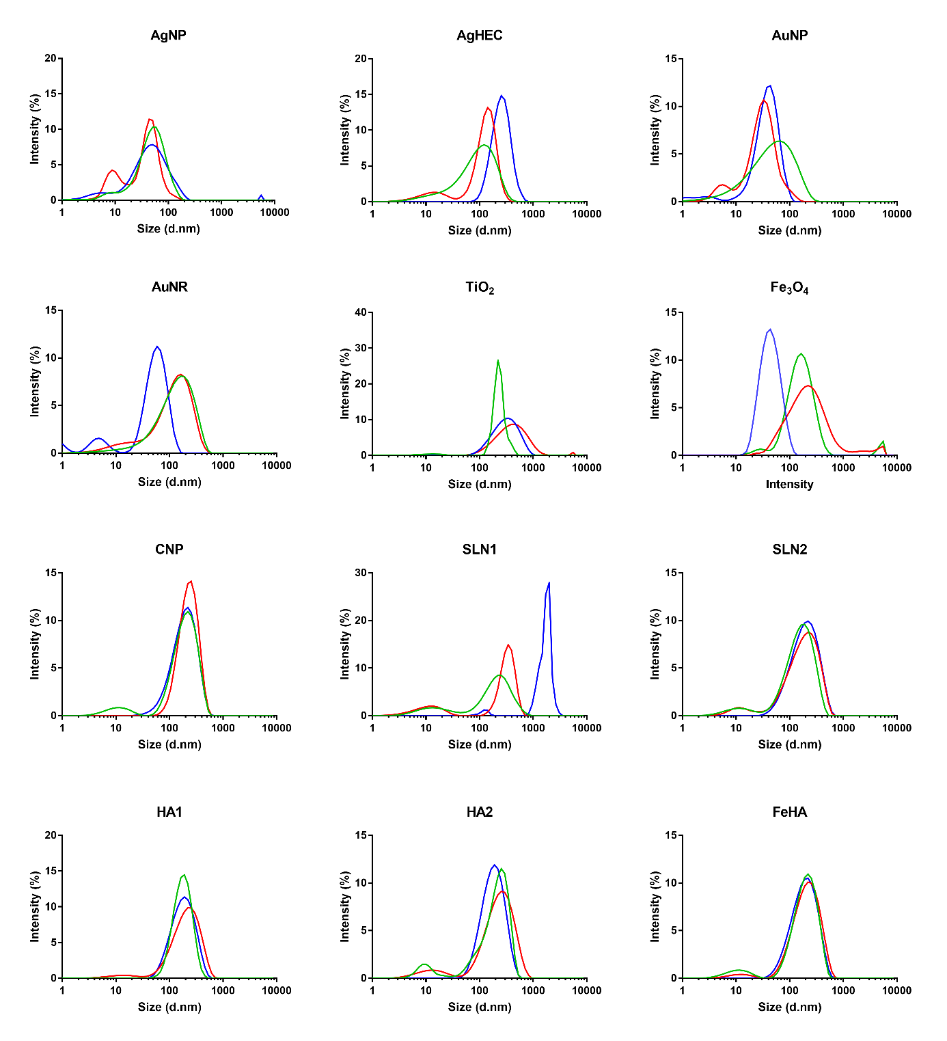


Figure S4. Size distribution of the NBMs. Size distributions (hydrodynamic diameter) in ultrapure water (blue line), complete cell culture medium immediately after dilution (red line) and complete cell culture medium after 24 h of incubation at 37°C, 5% CO2 (green line). Size dis-tributions were measured using a nanoZS zetasizer (Malvern instruments), with 100 µg/mL NBMs prepared using the same procedure as for cell exposure.


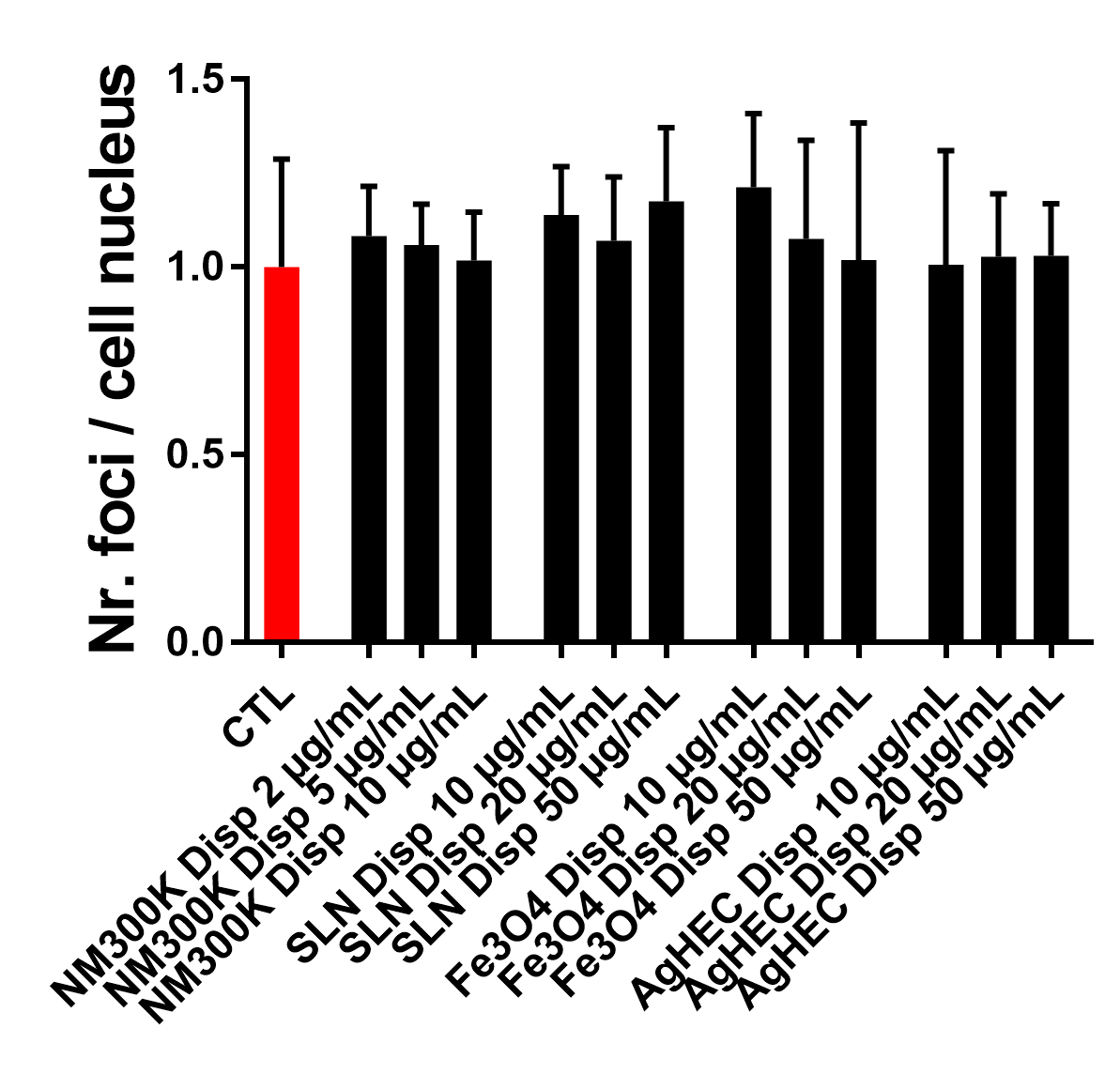


Figure S5. 53BP1 assay performed with the surfactants used for NBM dispersion. Three concentrations of surfactant were tested, which correspond to the volume of surfactant present in NBM suspensions when NBM concentration is 2, 5 or 10 µg/mL (NM300K) or 10, 20 or 50 µg/mL (SLN, Fe3O4, AgHEC). The graph reports the number of foci per cell nucleus, normalized to the number of 53BP1 foci in control (unexposed) cells. Average ± standard deviation of three independent experiments with five technical replicates (wells) per experiment, and 500 cells counted in each of the measured well.

**
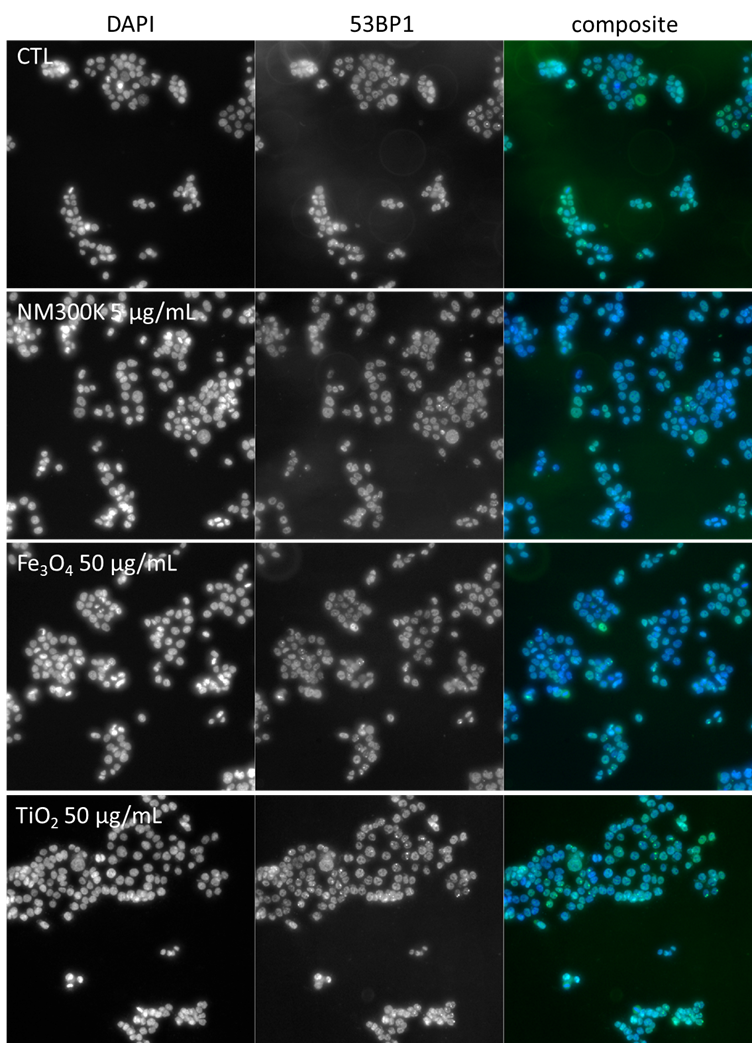
**

Figure S6. Typical images obtained in the 53BP1 assay on NMs and NBMs. Images were recorded on HCT116 cells exposed for 24 h to 5 µg/mL NM300K or to 50 µg/mL Fe3O4 or TiO2, and then immunostained with a 53BP1 antibody (green) and counterstained with Hoechst 33342 (blue, cell nucleus). The composite image is obtained by merging Hoechst 33342 and 53BP1 image, with artificial blue and green colours.


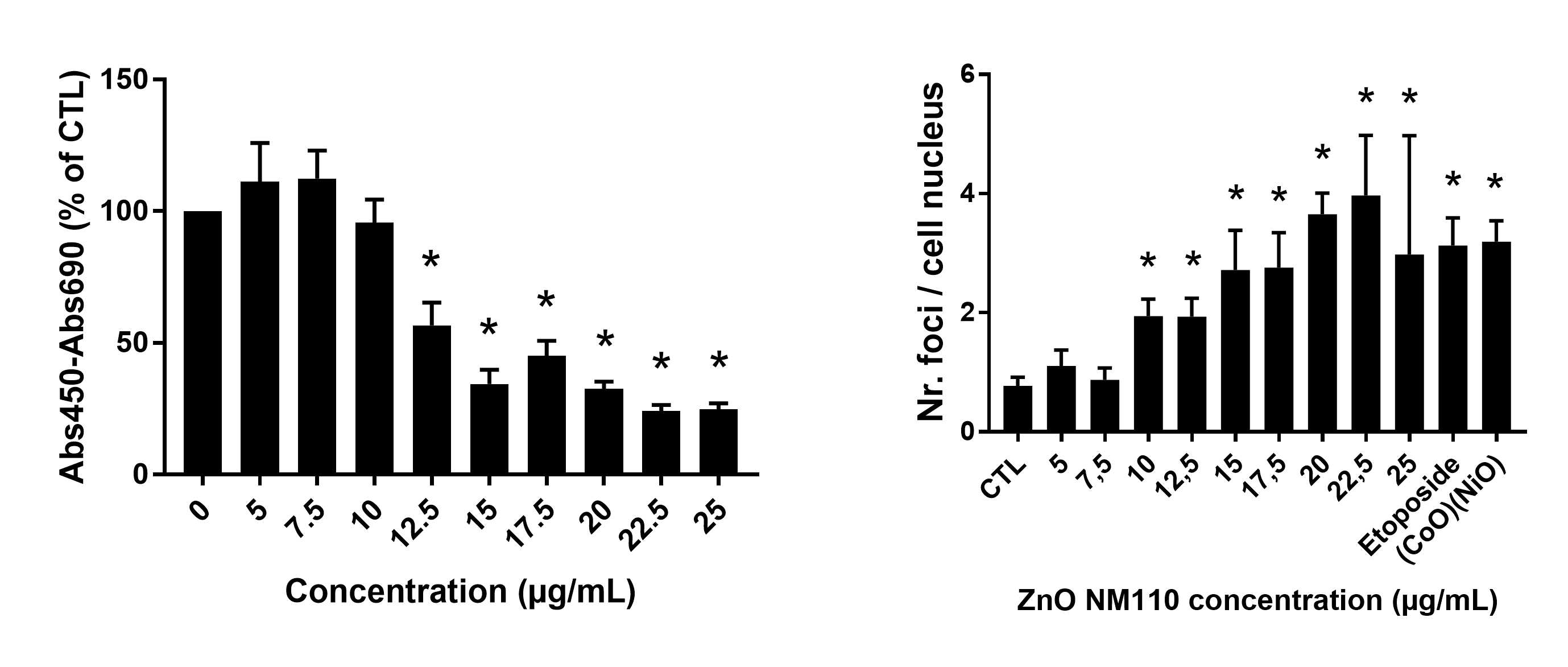


Figure S7. Cytotoxicity and induction of 53BP1 foci by the NM110 ZnO benchmark material. NM110 was provided by the JRC repository, and tested at concentration ranging from 5 to 25 µg/mL (exposure for 24 h). Impact on cell viability was assessed using the WST-1 assay, and DNA damaging potential via the 53BP1 assay.

**References**

1. Farcal, L.; Andon, F.T.; Di Cristo, L.; Rotoli, B.M.; Bussolati, O.; Bergamaschi, E.; Mech, A.; Hartmann, N.B.; Rasmussen, K.; Riego-Sintes, J.; et al. Comprehensive In Vitro Toxicity Testing of a Panel of Representative Oxide Nanomaterials: First Steps towards an Intelligent Testing Strategy. PloS one 2015, 10, doi:10.1371/journal.pone.0127174.

2. Totaro, S.; Cotogno, G.; Rasmussen, K.; Pianella, F.; Roncaglia, M.; Olsson, H.; Riego Sintes, J.M.; Crutzen, H.P. The JRC Nanomaterials Repository: A unique facility providing representative test materials for nanoEHS research. Regulatory toxicology and pharmacology : RTP 2016, 81, 334-340, doi:10.1016/j.yrtph.2016.08.008.

3. Costa, A.L.; Blosi, M. 2016. Patent nr. PCT/IB2016/050501.

4. Costa, A.L.; Blosi, M.; Brigliadori, A.; Zanoni, I.; Ortelli, S.; Simeone, F.C.; Delbue, S.; D'Alessandro, S.; Parapini, S.; Vineis, C.; et al. Eco design for Ag-based solutions against SARS-CoV-2 and E. coli. Environ. Sci.-Nano 2022, 9, 4295-4304, doi:10.1039/d2en00178k.

5. D'Elios, M.M.; Aldinucci, A.; Amoriello, R.; Benagiano, M.; Bonechi, E.; Maggi, P.; Flori, A.; Ravagli, C.; Saer, D.; Cappiello, L.; et al. Myelin-specific T cells carry and release magnetite PGLA-PEG COOH nanoparticles in the mouse central nervous system. RSC advances 2018, 8, 904-913, doi:10.1039/c7ra11290d.

6. Nikoobakht, B.; El-Sayed, M.A. Preparation and growth mechanism of gold nanorods (NRs) using seed-mediated growth method. Chemistry of Materials 2003, 15, 1957-1962, doi:10.1021/cm020732l.

7. Sau, T.K.; Murphy, C.J. Seeded high yield synthesis of short Au nanorods in aqueous solution. Langmuir 2004, 20, 6414-6420, doi:10.1021/la049463z.

8. Wijaya, A.; Hamad-Schifferli, K. Ligand customization and DNA functionalization of gold nanorods via round-trip phase transfer ligand exchange. Langmuir 2008, 24, 9966-9969, doi:10.1021/la8019205.

9. Joint Research, C.; Institute for, H.; Consumer, P.; Gaillard, C.; Mech, A.; Motzkus, C.; Gilliland, D.; Rasmussen, K.; Rousset, D.; Shivachev, B.; et al. Titanium dioxide, NM-100, NM-101, NM-102, NM-103, NM-104, NM-105 : characterisation and physico-chemical properties; Publications Office: 2014.

10. Baldi, G.; Bitossi, M.; M., C.F.; Ravagli, C.; D'Elios, M.; Benagiano, M. Magnetic nanoparticles functionalized with cathecol, production and use thereof. 2015.

11. You, L.; Liu, X.; Fang, Z.; Xu, Q.; Zhang, Q. Synthesis of multifunctional Fe(3)O(4)@PLGA-PEG nano-niosomes as a targeting carrier for treatment of cervical cancer. Materials science & engineering. C, Materials for biological applications 2019, 94, 291-302, doi:10.1016/j.msec.2018.09.044.

12. Joint Research, C.; Institute for, H.; Consumer, P.; Waegeneers, N.; Van Steen, F.; Petrov, O.; Bau, S.; Verleysen, E.; Van Doren, E.; Thieriet, N.; et al. Multi-walled carbon nanotubes, NM-400, NM-401, NM-402, NM-403, characterisation and physico-chemical properties; Publications Office: 2014.

13. Guimarães, B.; Gomes, S.I.L.; Campodoni, E.; Sandri, M.; Sprio, S.; Blosi, M.; Costa, A.L.; Amorim, M.J.B.; Scott-Fordsmand, J.J. Environmental Hazards of Nanobiomaterials (Hydroxyapatite-Based NMs)-A Case Study with Folsomia candida-Effects from Long Term Exposure. Toxics 2022, 10, doi:10.3390/toxics10110704.

14. Tampieri, A.; D'Alessandro, T.; Sandri, M.; Sprio, S.; Landi, E.; Bertinetti, L.; Panseri, S.; Pepponi, G.; Goettlicher, J.; Banobre-Lopez, M.; et al. Intrinsic magnetism and hyperthermia in bioactive Fe-doped hydroxyapatite. Acta Biomaterialia 2012, 8, 843-851, doi:10.1016/j.actbio.2011.09.032.

15. Campodoni, E.; Montanari, M.; Artusi, C.; Bassi, G.; Furlani, F.; Montesi, M.; Panseri, S.; Sandri, M.; Tampieri, A. Calcium-Based Biomineralization: A Smart Approach for the Design of Novel Multifunctional Hybrid Materials. Journal of Composites Science 2021, 5, 278.

16. Roveri, N.; Falini, G.; Sidoti, M.C.; Tampieri, A.; Landi, E.; Sandri, M.; Parma, B. Biologically inspired growth of hydroxyapatite nanocrystals inside self-assembled collagen fibers. Materials Science & Engineering C-Biomimetic and Supramolecular Systems 2003, 23, 441-446, doi:10.1016/s0928-4931(02)00318-1.

17. Sprio, S.; Sandri, M.; Iafisco, M.; Panseri, S.; Adamiano, A.; Montesi, M.; Campodoni, E.; Tampieri, A. Bio-inspired assembling/mineralization process as a flexible approach to develop new smart scaffolds for the regeneration of complex anatomical regions. Journal of the European Ceramic Society 2016, 36, 2857-2867, doi:https://doi.org/10.1016/j.jeurceramsoc.2016.01.005.
