# Supplementary figures and images for: Nanomaterial genotoxicity evaluation using the high-throughput p53-binding protein 1 (53BP1) assay
Source: PLoS One. 2023 Sep 15;18(9):e0288737. doi: 10.1371/journal.pone.0288737 (PMC10503773; doi:10.1371/journal.pone.0288737)

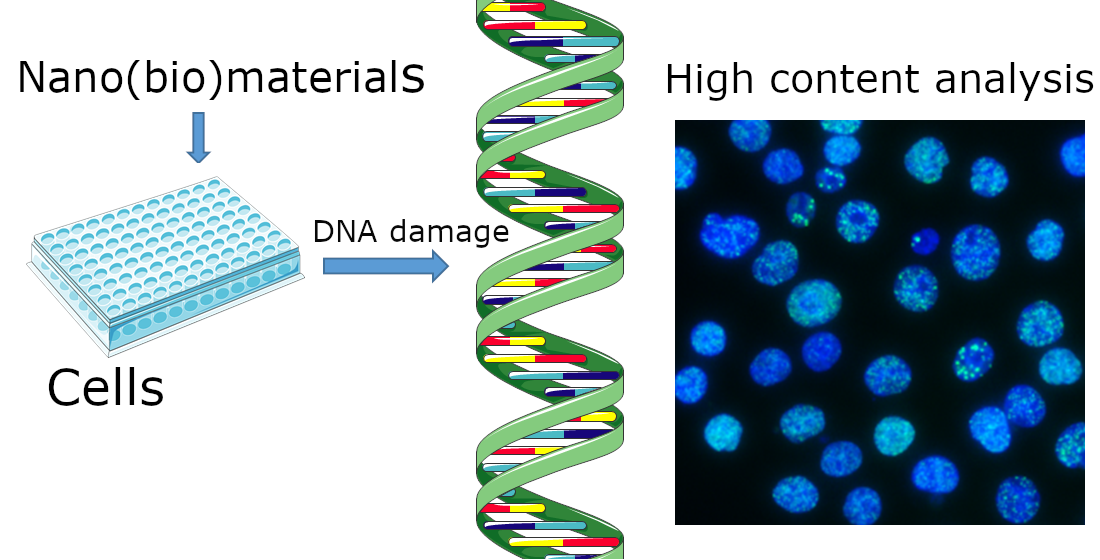

Supplement: S1 Graphical abstract — (PNG) [file pone.0288737.s002.png]
